# Machine learning model to predict mental health crises from electronic health records

**DOI:** 10.1038/s41591-022-01811-5

**Published:** 2022-05-16

**Authors:** Roger Garriga, Javier Mas, Semhar Abraha, Jon Nolan, Oliver Harrison, George Tadros, Aleksandar Matic

**Affiliations:** 1 Koa Health, Barcelona, Spain; 2 Universitat Pompeu Fabra, Department of Information and Communication Technologies, Barcelona, Spain; 3 Kannact, Barcelona, Spain; 4 Birmingham and Solihull Mental Health NHS Foundation Trust, Birmingham, UK; 5 University of Warwick, Warwick, UK; 6 Aston Medical School, Aston University, Aston, UK

**Keywords:** Health care, Psychology, Predictive markers

## Abstract

The timely identification of patients who are at risk of a mental health crisis can lead to improved outcomes and to the mitigation of burdens and costs. However, the high prevalence of mental health problems means that the manual review of complex patient records to make proactive care decisions is not feasible in practice. Therefore, we developed a machine learning model that uses electronic health records to continuously monitor patients for risk of a mental health crisis over a period of 28 days. The model achieves an area under the receiver operating characteristic curve of 0.797 and an area under the precision-recall curve of 0.159, predicting crises with a sensitivity of 58% at a specificity of 85%. A follow-up 6-month prospective study evaluated our algorithm’s use in clinical practice and observed predictions to be clinically valuable in terms of either managing caseloads or mitigating the risk of crisis in 64% of cases. To our knowledge, this study is the first to continuously predict the risk of a wide range of mental health crises and to explore the added value of such predictions in clinical practice.

## Abstract (web summary)

Machine learning applied on electronic health records can predict mental health crises 28 days in advance and become a clinically valuable tool for managing caseloads and mitigating the risk of crisis.

## Main

Nearly 1 billion people worldwide live with a mental disorder^1^. With the global mental health emergency considerably exacerbated by the Coronavirus Disease 2019 pandemic, healthcare systems face a growing demand for mental health services coupled with a shortage of skilled personnel^2–5^. In clinical practice, considerable demand arises from mental health crises—that is, situations in which patients can neither care for themselves nor function effectively in the community and situations in which patients may hurt themselves or others^6,7^. Timely treatment can prevent exacerbating the symptoms that lead to such crises and subsequent hospitalization^8^. However, patients are frequently already experiencing a mental health crisis when they access urgent care pathways as their primary entry point to a hospital or psychiatric facility. By this point, it is too late to apply preventative strategies, limiting the ability of psychiatric services to properly allocate their limited resources ahead of time. Therefore, identifying patients at risk of experiencing a crisis before its occurrence is central to improving patient outcomes and managing caseloads^9^.

In busy clinical settings, the manual review of large quantities of data across many patients to make proactive care decisions is impractical, unsustainable and error-prone^10^. Thus, shifting such tasks to the automated analysis of electronic health records (EHRs) holds great promise to revolutionize health services by enabling large-scale continuous data review. Research has already demonstrated the feasibility of predicting critical events associated with a wide range of healthcare problems, including hypertension, diabetes, circulatory failure, hospital readmission and in-hospital death^11–17^. However, the mental health literature is limited to predicting specific types of events—such as suicide, self-harm and first episode psychosis^18–28^—rather than continuously predicting the breadth of mental health crises that require urgent care or hospitalization. Much remains unknown about the feasibility of querying machine learning models continuously to estimate the risk of an imminent mental health crisis. This would enable optimizing healthcare staff allocation and preventing crisis onset. Furthermore, even a highly accurate predictive model does not guarantee improved mental health outcomes or long-term cost savings^29,30^; therefore, it remains unclear whether new predictive technologies could provide tools that are useful to mental healthcare practitioners^31,32^.

This research explores the feasibility of predicting any mental health crisis event, regardless of its cause or the underlying mental disorder, and we investigate whether such predictions can provide added value to clinical practice. The underpinning assumption is that there are historical patterns that predict future mental health crises and that such patterns can be identified in real-world EHR data, despite its sparseness, noise, errors and systematic bias^33^. To this end, we developed a mental crisis risk model by inputting EHR data collected over 7 years (2012–2018) from 17,122 patients into a machine learning algorithm. We evaluated how accurately the model continuously predicted the risk of a mental health crisis within the next 28 days from an arbitrary point in time, with a view to supporting dynamic care decisions in clinical practice. We also analyzed how the model’s performance varied across a range of mental health disorders, across different ethnic, age and gender groups and across variations in data availability. Furthermore, we conducted a prospective cohort study to evaluate the crisis prediction algorithm in clinical practice from 26 November 2018 to 12 May 2019. The crisis predictions were delivered on a biweekly basis to four different groups of clinicians (in total, 60 clinicians attending 1,011 cases over 6 months), who evaluated whether and how such predictions helped them manage caseload priorities and mitigate the risk of crisis.

## Results

### Prediction target

**Fig. 1:**
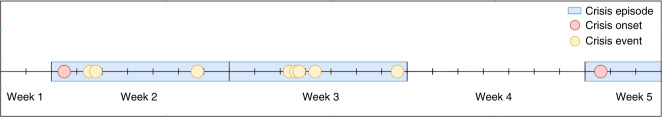
Crisis episode example. Example of a crisis episode timeline: crisis onset is the first crisis event of a crisis episode that follows a stable week (that is, a week without crisis events).

As our main goal was to develop a predictive tool that could help healthcare workers manage caseload priorities and pre-emptively intervene to mitigate the risk of crisis, we established the prediction target to align with the service-oriented approach to defining crisis^7^—that is, the onset of severe symptoms that require substantial healthcare resources. Notwithstanding a wide range of approaches to defining a mental health crisis in the literature (namely service-oriented, risk-focused, self-defined and negotiated definitions^7^), these definitions consistently describe an event that substantially affects the life of a patient and the load on healthcare services. Correspondingly, our dataset included crisis events, which were registered every time a patient urgently needed mental health crisis services, such as emergency assessment, inpatient admission, home treatment assessment or hospitalization. Because crisis events frequently occur in succession when a patient is undergoing a crisis, predicting each singular crisis event registered in the EHR would be of little clinical relevance because patients who experience one crisis event receive close clinical attention over successive days. Therefore, we defined the prediction target as the onset of a crisis episode, which contains one or more crisis events, preceded by at least one full stable week without any crisis event (Fig. 1). Accordingly, we trained the machine learning model to predict the onset of a crisis episode—that is, the first crisis event in an episode—within the next 28 days. The time horizon of 28 days was selected based on input from clinicians to support the management of caseload priorities and to enable pre-emptive interventions. Notably, using different time horizons (that is, other than 28 days) or defining a stable period before a relapse other than 7 days did not substantially affect the model’s performance (Supplementary Table 9).

### Dataset

**Extended Data Fig. 1:**
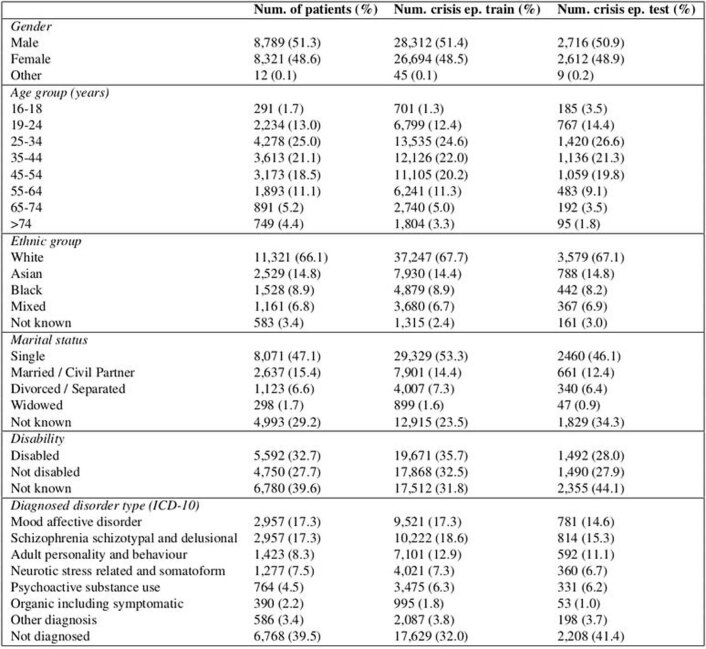
Demographics and patient’s characteristics. Summary of the retrospective cohort per gender, age group, ethnic group, marital status and primary diagnosed disorder category; including the number and percentage of patients, crisis episodes in train and test per each group category. No major differences in the distribution of crisis episodes in train and test were observed between group categories.

**Extended Data Fig. 2:**
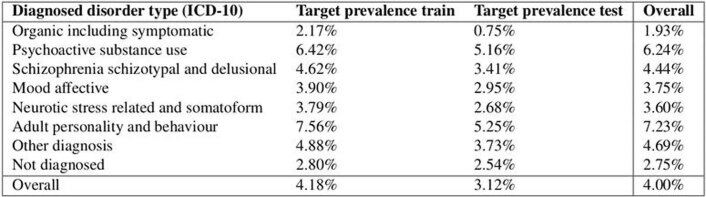
Prevalence of the target variable (a crisis episode within the next 28 days) per disorder type. Prevalence of the target variable for different disorders. Adult personality and behaviour (F6 in ICD-10 categorisation) and Psychoactive substance use (F1 in ICD-10 categorisation) show a slightly greater prevalence of crisis episodes, whereas the prevalence was lower for Organic including symptomatic mental disorders (F0 in ICD-10 categorisation) and Not diagnosed patients. A small difference was observed between train and test, with a lower prevalence in the test set overall.

Upon applying the exclusion criteria (Methods), the study cohort data contained 5,816,586 records collected between September 2012 and November 2018 from 17,122 unique patients aged between 16 and 102 years. This included patients with a wide range of diagnosed disorders, including mood, psychotic, organic, neurotic and personality disorders. The two genders and the full range of ethnic groups were well represented in the dataset (51.5% males and 48.6% females; 66% White, 15% Asian, 9% Black and 7% Mixed). No major deviations were observed in the crisis distribution according to gender or ethnicity or disability (see Extended Data Fig. 1 for the complete summary). In total, 60,388 crisis episodes were included in the analysis, with a mean of 24 crisis events per episode. Among the 1,448,542 crisis events that were recorded, 942,017 corresponded to hospitalizations. The rest of the EHR data included phone and in-person contact with patients (2,239,632 records), referrals (250,864 records) and well-being and risk assessments (118,255 and 248,629 records) (see Supplementary Table 1 for more details). Our prediction target variable had a prevalence of 4.0% on average across the entire dataset, varying from 1.9% (organic disorders) to 7.2% (disorders of adult personality and behavior) (see Extended Data Fig. 2 for a detailed breakdown by diagnosis, training and test sets).

### Development of a mental health crisis prediction model

**Fig. 2:**
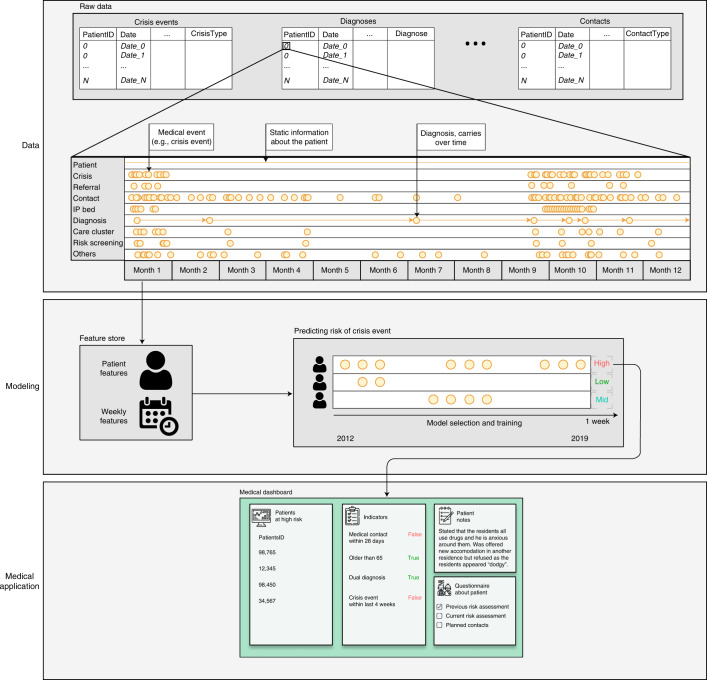
System diagram. Time series of events are represented with the timestamps and event characteristics in different SQL tables in the hospital’s database. These tables are processed and converted into features for the modeling task. Models are trained, tuned and selected based on the data for the period 2012–2019. The system predicts the risk of crisis onset within the next 28 days (whereby the algorithm is queried every week for every patient). The patients with the highest predicted risk are displayed on the dashboard delivered to clinicians alongside key indicators, patient notes and a questionnaire form about each patient, which the clinician fills out. The icons in this figure were made by Freepik from www.flaticon.com. IP, inpatient.

The model was designed to be queried weekly to infer each patient’s risk of experiencing a crisis episode during the upcoming 28-day period. To build the model, we extracted three feature categories: (1) static or semi-static patient information (such as age, gender and International Classification of Diseases 10 (ICD-10)^34^ coded diagnoses); (2) latest available assessments and interactions with the hospital (for example, most recent risk assessments or well-being indicators and severity and number of crisis events in the last episode and similar); and (3) variables representing the time elapsed since the registered events (for example, crisis episodes, contacts and referrals). In total, we extracted 198 features (Supplementary Table 5). When the system was implemented, instead of a binary outcome, the model was generating a predicted risk score (PRS) between 0 and 1 for each patient. Figure 2 presents the end-to-end process.

**Fig. 3:**
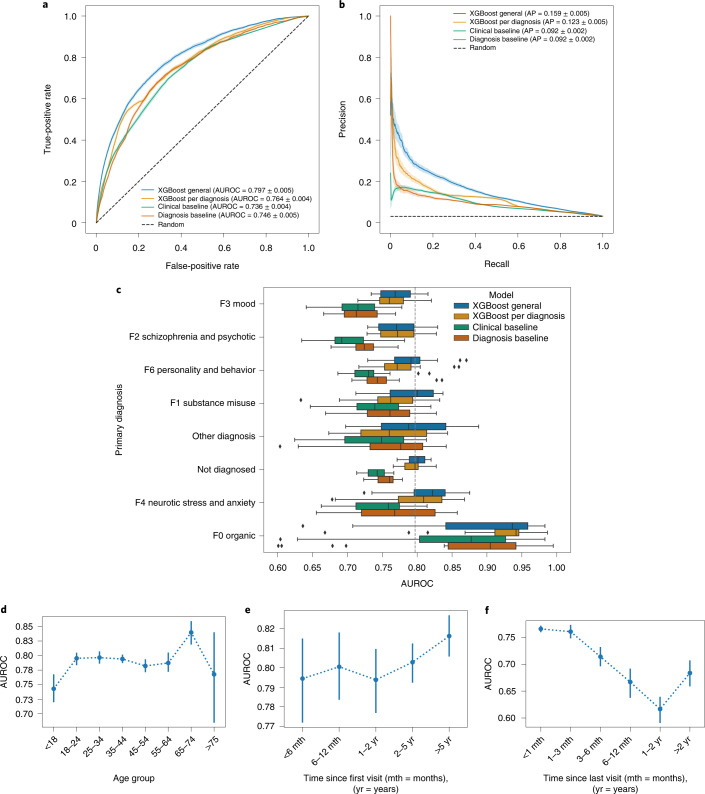
Final model performance. **a**, ROC curve for the crisis prediction task. Comparison among the proposed final model (XGBoost general), a proposed diagnosis-specific model (XGBoost per diagnosis) and two baseline models. The solid lines and lighter-colored envelopes around each line were derived from the test evaluations (*n* = 25) as the mean and 95% CI, respectively. **b**, Precision-recall curve for the crisis prediction task with the same characteristics as **a**. **c**, Box plot of the AUROC curve evaluated per diagnosis. Comparison among the four models considered as in **a** and **b**. The solid line corresponds to the median value; the box limits correspond to the first Q1 (left limit) and third Q3 (right limit) quartiles; the whiskers denote the rest of the distribution range from Q1–1.5 (Q3–Q1) (left whisker) to Q3 + 1.5 (Q3–Q1) (right whisker); and the points displayed correspond to the outliers. **d**–**f**, AUROC curve evaluated for different subsets of the study cohort based on age group (**d**), time since the patient first visited the hospital (**e**) and time since the patient’s last crisis episode (**f**). The dots and bars derive from the test evaluations (*n* = 25) as the mean and 95% CI, respectively.

**Extended Data Fig. 3:**
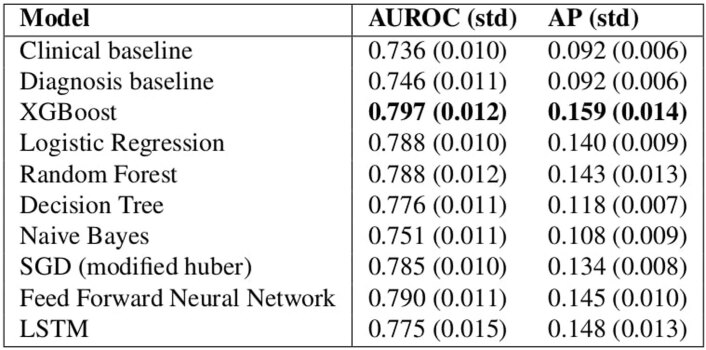
Evaluation of multiple Machine Learning models to predict the risk of crisis onset during the following 28 days. Values in bold denote the model with the highest performance.

**Extended Data Fig. 4:**
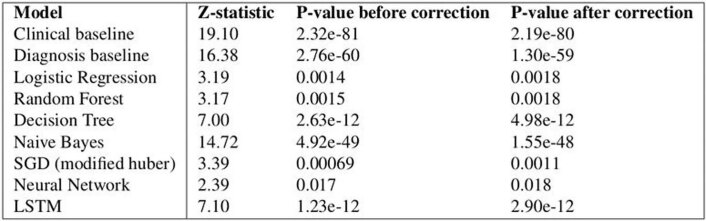
Statistical significance analysis comparing the AUROC of XGBoost to the other models. Statistical significance analysis was done using the Mann-Whitney U test. The two-stage step-up method of Benjamini, Krieger and Yekutieli was used to correct the p-values of the multiple tests performed.

**Extended Data Fig. 5:**
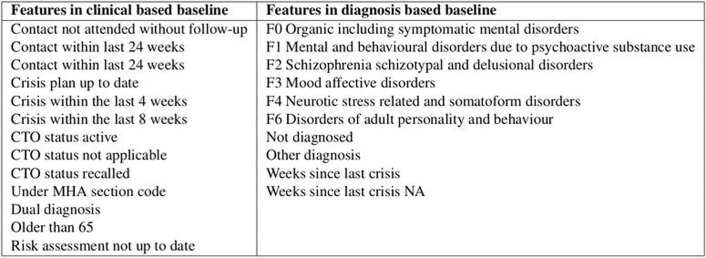
Features used in each baseline.

**Extended Data Fig. 6:**
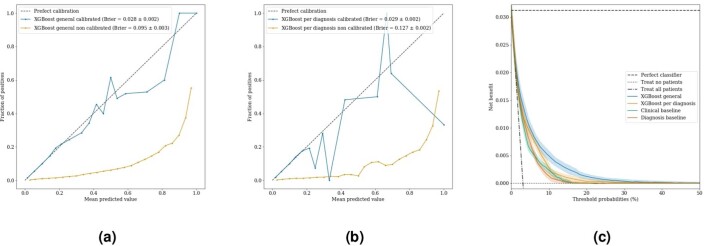
Model calibration and net benefit. **(a)**, **(b)** Calibration curves of the general model (XGBoost general) and diagnosis specific models (XGBoost per diagnosis). Yellow and blue lines represent the non calibrated and calibrated curves for both models, respectively. The diagonal dotted line shows the ideal calibration reference curve. **(c)** Decision curve shows the net benefit versus the threshold probability, for the proposed models and baselines. The general model (XGBoost general) outperforms the baselines and the diagnosis specific model (XGBoost per diagnosis) at all thresholds. The solid lines and lighter-coloured envelopes around each line were derived from the test evaluations (n = 25) as the mean and 95% confidence interval respectively.

We tested a range of machine learning techniques, including decision trees, probabilistic, ensembles and deep learning-based classifiers. Consistent with similar studies^11,16,35^, XGBoost (eXtreme Gradient Boosting) outperformed most of the other methods evaluated (although, in some cases, only by small margins). The Mann–Whitney *U*-test suggested a significantly better performance of XGBoost (*P* < 0.01) when compared to the other methods, except for a feed-forward neural network (Extended Data Figs. 3 and 4). The XGBoost model relied on an automatically selected subset of 104 features to predict mental health crises for all patients in our dataset (referred to as the general model). We benchmarked this model against two baseline classifiers: (1) the clinical-practice-based baseline model, developed to emulate a doctor’s decisions (specifically, a decision tree using a selection of patient status indicators that doctors in our clinical setting use to assess the risk of relapse); and (2) the diagnosis-based baseline model, developed as a logistic regression that relies solely on diagnosis and time elapsed since the last crisis, resembling a threshold-based rule system (see Extended Data Fig. 5 for each baseline’s list of features). The area under the receiver operating characteristic (AUROC) curves of the general model, the clinical-practice-based baseline and the diagnosis-based baseline were 0.797 (95% confidence interval (CI) 0.793–0.802), 0.736 (95% CI 0.733–0.740) and 0.746 (95% CI 0.741–0.750) (Fig. 3). For unbalanced datasets, as in our case, the average precision (AP)^36^ represents a more informative metric^37^, and the APs obtained for the general model, the clinical-practice-based baseline and the diagnosis-based baseline were 0.159 (95% CI 0.154–0.165), 0.092 (95% CI 0.090–0.094) and 0.092 (95% CI 0.089–0.094). The general model significantly outperformed the two baseline models (*P* < 0.0001 for both AUROC and AP). We calibrated the predictions using isotonic regression^38^ (Extended Data Fig. 6), ensuring that the predicted risk reflected the actual expected risk of experiencing a crisis episode^39^, and obtained a Brier score^40^ of 0.028 (95% CI 0.028–0.029). Additionally, the general model demonstrated a more substantial net benefit in the decision curve analysis^41^ than the baseline models and default strategies (Extended Data Fig. 6).

### Model performance for different disorders

**Extended Data Fig. 7:**
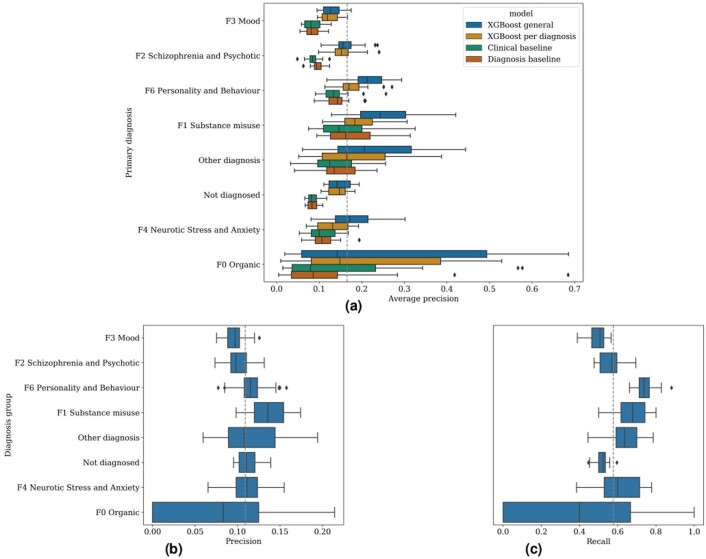
Model performance per diagnosis. **(a)** Box-plot of the average precision (area under the precision recall curve) evaluated per diagnosis. Comparison between the final model (XGBoost general), a diagnosis specific model (XGBoost per diagnosis) and two baseline models. Dotted line marks the mean average precision of the general model (n=25).**(b)**, **(c)** Box-plot of the precision and recall respectively per diagnosis with a threshold corresponding to 15% of false positive rate (obtained with evaluationson patients in the test set (n=25)). **(c)** Box-plot of the recall per diagnosis with a threshold corresponding to 15% of false positive rate. In all Box-plots the solid line corresponds to the median value, the box limits to the first Q1 (left limit) and third (right limit) quartiles, the whiskers denote the rest of the distribution range from Q1-1.5(Q3-Q1) (left whisker) to Q3+1.5(Q3-Q1) (right whisker) and the points displayed correspond to the outliers.

We evaluated the performance of the prediction model in patients with mental health disorders grouped according to the first-level categorization of the ICD-10 (ref. ^34^). We relied solely on AUROC to evaluate the model performance of each disorder because the AP is an inappropriate metric for comparing groups with different prevalence values^37^. The general model performed considerably better for organic disorders, with an AUROC of 0.890 (95% CI 0.852–0.928) compared to the overall performance of 0.797 (95% CI 0.793–0.802). For other diagnostic groups, the performance ranged between 0.770 (95% CI 0.760–0.779) and 0.814 (95% 0.796–0.831). The lowest performance was observed for mood-affective disorders, followed by schizophrenia and schizotypal and delusional disorders. Separate models for each diagnosis subgroup were developed and compared to the general model. The general model consistently outperformed the baseline models, and no disorder-specific model performed significantly better than the general model (Fig. 3c and Extended Data Fig. 7).

### Model performance for different age groups

**Extended Data Fig. 8:**
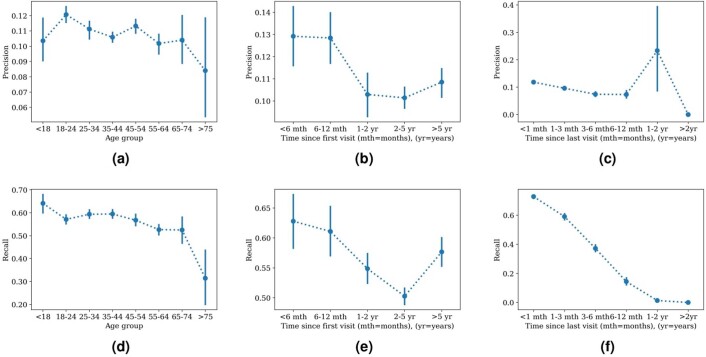
Precision and recall per cohort. Precision evaluated with respect to **(a)** different age groups; **(b)** time since the first hospital visit; **(c)** time since the last hospital visit; with a threshold corresponding to 15% of false positive rate. Recall evaluated with respect to **(d)** different age groups; **(e)** time since the first hospital visit; **(f)** time since the last visit; with a threshold corresponding to 15% of false positive rate obtained with evaluations in the test set (n=25). The dots and bars were derived from the test evaluations (n = 25) as the mean and 95% confidence interval respectively.

We evaluated the general model in subgroups of patients across different age groups. The model performance dropped to 0.743 (95% CI 0.718–0.767) for patients younger than 18 years and increased to 0.840 (95% CI 0.820–0.859) for patients aged between 65 and 74 years. For the other age groups, the model performed similarly, with an AUROC between 0.782 (95% CI 0.771–0.793) and 0.796 (95% CI 0.786–0.806) (Fig. 3d and Extended Data Fig. 8).

### Effect of data availability on model performance

Data availability substantially affected model performance. For example, if there was no information about a patient for 1 year or more, the AUROC dropped to 0.617 (95% CI 0.592–0.641). Meanwhile, for patients who had at least one record within the previous month, the AUROC was 0.765 (95% CI 0.761–0.771). A longer history of patient data in the EHR of the hospital improved the model’s performance, with AUROCs ranging from 0.794 (95% CI 0.772–0.817) for patients who had first visited within the previous 6 months to 0.816 (95% CI 0.805–0.827) for patients whose first record dated back 5 years or more (Fig. 3e,f and Extended Data Fig. 8).

### Analysis of the most predictive features

**Fig. 4:**
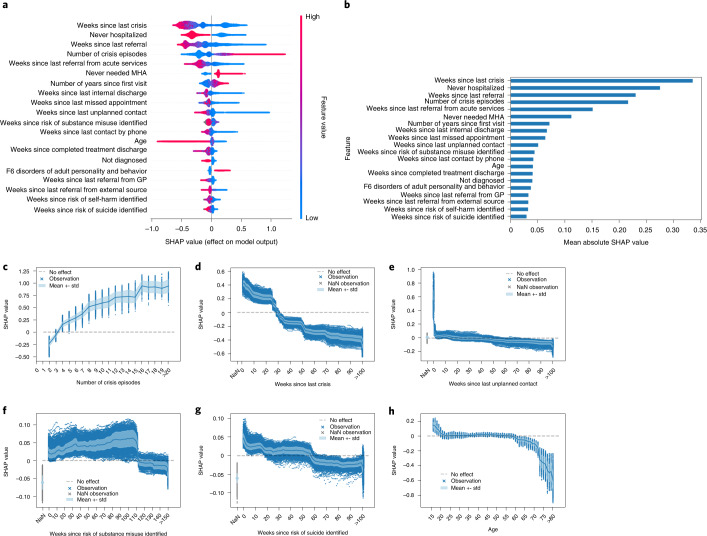
Most predictive features. **a**, Complete distribution of the SHAP values for the top 20 features based on the highest mean absolute SHAP value. Each sample of the test set is represented as a data point per feature, and the *x* axis shows the positive or negative effect on the model’s prediction of the feature. The color coding depicts the value of the feature and is scaled independently based on the range observed in the data. **b**, Absolute feature contribution of the 20 features with the highest mean absolute SHAP value. **c**–**h**, Six examples of dependence plots showing the effect on the PRS with respect to the feature value. Each data point (*n* = 371,010) represents a sample in the test set, with the solid lines and the lighter-colored envelopes representing, respectively, the mean effect and its standard deviation per feature value. The variability at each feature value corresponds to interaction with the rest of the features. Missing values (representing the absence of events) are colored gray. GP, general practitioner; MHA, mental health act; std, standard deviation.

We analyzed the relative effect of the top 20 features on the model at each data point in the test set according to the mean absolute SHAP (SHapley Additive exPlanations)^42^ value (Fig. 4). The historical severity of symptoms (specifically, the total number of crisis episodes and the duration of the last episode), interactions with the hospital (including unplanned contacts, missed appointments or a recent crisis), patient characteristics (including age and individual risk indices) and total time since the patient’s first hospital visit carried most of the general model’s predictive power (Fig. 4a,b).

To further examine the effect of each variable, we also analyzed the SHAP values of the top 20 features separately (Fig. 4c–h and Supplementary Fig. 1). The recency of records (especially important events such as crises and unplanned contacts) had a major effect on the PRS, positively contributing to the risk score up to a threshold value beyond which they began driving the risk score down. However, the effect of different events varied over time, with some having a long-lasting effect and others affecting the risk score only during the first weeks after their incidence. Unplanned contacts with a patient had the biggest short-term effect, but their effect disappeared almost completely after only 2 weeks. Longer-lasting effects were observed for events encoding contacts with the carer and missed appointments, which produced sustained effects on the PRS for 10 weeks and 16 weeks. In turn, referrals and crises considerably affected the PRS both positively (for approximately 6 months or 25 weeks and 29 weeks, respectively) and negatively (thereafter). The variables reflecting severe symptoms generally demonstrated the longest-lasting effects on the PRS. For example, referrals from acute services, positive suicide risk assessments and positive substance misuse assessments affected the PRS for 1–2 years. In most cases, the presence of important events was associated with a previous clinical deterioration, which means that the absence of certain types of events—denoted in the features by NaN (not a number) values—suggests less severe symptoms in the patient’s history and negatively affected the PRS. Consider, for instance, a patient who had never been hospitalized. This had a negative influence on the PRS. In contrast, a positive influence would be observed for a patient who had been hospitalized at least once before.

**Extended Data Fig. 9:**
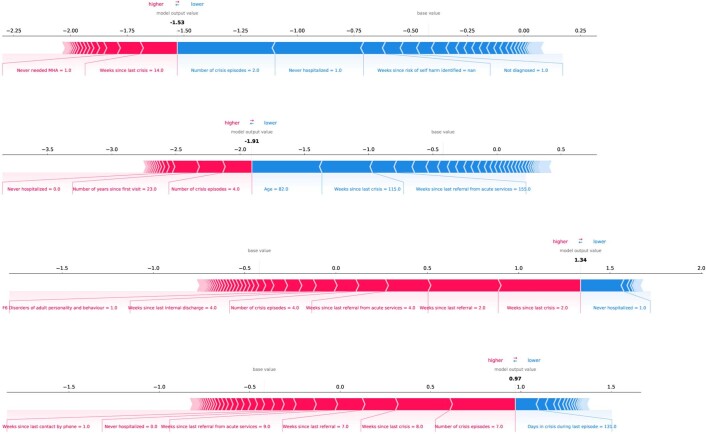
Examples of features contribution to the predicted risk score. Four representative force plots, depicting how the features contributed to the prediction for four specific data points. From top to bottom: Patient not going to have a crisis during the next four weeks (target=0), the model assigned a prediction value of 0.178. Patient not going to have a crisis during the next four weeks (target=0), the model assigned a prediction value of 0.129. Patient going to have a crisis during the next four weeks (target=1) the model assigned a prediction value of 0.792. Patient going to have a crisis during the next four weeks (target=1) the model assigned a prediction value of 0.725.

Finally, to investigate the complexity of the interactions among features that drive the PRS, we used the force plots of positive and negative predictions (Extended Data Fig. 9). The sign and magnitude of each variable’s contribution differed according to the value of the other variables and its own value, thus demonstrating the model’s complex and non-linear nature.

### Clinical evaluation

To assess the added value of the algorithm in clinical practice, we conducted a prospective study in which crisis predictions were delivered to clinicians every 2 weeks. We queried our prediction model to rank patients in descending order based on the PRS. Four multidisciplinary clinical teams (Community Mental Health Teams (CMHTs); see Table 1 for the team composition) each received a dashboard displaying the 25 patients with the highest PRS. Before exploring the algorithm’s practical value, we asked the CMHTs to assess the risk of crisis for each patient and rate their agreement with each prediction. Disagreement was recorded in 7% (*n* = 65) of all the presented predictions provided over 6 months, ranging from 3% (*n* = 6) to 12% (*n* = 27) across the four CMHTs. Overall, CMHTs rated 38% (*n* = 351) of the cases as low risk, 44% (*n* = 407) as medium risk, 13% (*n* = 119) as high risk and less than 0.1% (*n* = 3) as being at imminent risk of experiencing a mental health crisis. Meanwhile, 6% (*n* = 55) of the reviewed cases were patients already experiencing a crisis. Upon reviewing the predictions, CMHTs responded that they would make contact either by telephone (5% of cases) or in person (8% of cases; average percentage calculated based on the responses of the four teams; see F1 in Table 2). This corresponds to patients who otherwise would not have been attended to. Although the predictions were accurate in most other cases, no further action was required because the CMHTs were already managing the risk.

**Table 1:** Prospective study participants and completion rate (grouped by team)

No. (%)	Team 1	Team 2	Team 3	Team 4	Total
Clinicians	*n* = 13	*n* = 19	*n* = 14	*n* = 14	*n* = 60
Male	5 (38)	6 (32)	5 (36)	4 (29)	20 (33)
Female	8 (62)	13 (68)	9 (64)	10 (71)	40 (67)
Nurses	12 (92)	15 (79)	11 (79)	13 (93)	51 (85)
Doctors	1 (8)	2 (11)	0 (0)	1 (7)	4 (7)
Occupational therapists	0 (0)	1 (5)	1 (7)	0 (0)	2 (3)
Duty workers	0 (0)	1 (5)	1 (7)	0 (0)	2 (3)
Social workers	0 (0)	0 (0)	1 (7)	0 (0)	1 (2)
Form completion	*n* = 292	*n* = 279	*n* = 196	*n* = 244	*n* = 1,011
F1	292 (100)	246 (87)	177 (90)	220 (89)	935 (92)
F2	274 (94)	221 (78)	159 (81)	202 (80)	856 (84)

**Table 2:** Responses to the feedback forms F1 and F2 from each team of clinicians involved in the prospective study

No. (%)	Team 1	Team 2	Team 3	Team 4	Total
F1 responses	*n* = 292	*n* = 246	*n* = 177	*n* = 220	*n* = 935
Assessment of patient’s risk of crisis					
Low risk	99 (34)	89 (36)	48 (27)	115 (52)	351 (38)
Medium risk	136 (47)	96 (39)	92 (52)	83 (38)	407 (44)
High risk	29 (10)	59 (24)	21 (12)	10 (5)	119 (13)
Imminent risk	2 (1)	0 (0)	1 (1)	0 (0)	3 (0)
Already in crisis	26 (9)	2 (1)	15 (8)	12 (5)	55 (6)
Have you taken / do you intend to take any actions as a result of this notification?					
Yes, contact to be made (Telf)	9 (3)	15 (6)	11 (6)	8 (4)	43 (5)
Yes, contact to be made (F2F)	12 (4)	38 (15)	11 (6)	10 (5)	71 (8)
No, contact made in last 7 days	46 (16)	28 (11)	41 (23)	29 (13)	144 (15)
No, risk already being managed	202 (69)	156 (63)	109 (61)	146 (66)	613 (65)
No, do not agree with assessment	23 (8)	9 (4)	6 (3)	27 (12)	65 (7)
F2 responses	*n* = 274	*n* = 221	*n* = 159	*n* = 202	*n* = 856
What is your current assessment of this patient’s condition?					
Low risk	110 (40)	102 (46)	47 (30)	110 (54)	369 (43)
Medium risk	124 (45)	72 (33)	83 (52)	73 (36)	352 (41)
High risk	25 (9)	42 (19)	16 (10)	7 (3)	90 (11)
Imminent risk	1 (0)	2 (1)	0 (0)	0 (0)	3 (0)
Already in crisis	14 (5)	3 (1)	13 (8)	12 (6)	42 (5)
Do you think that this additional information has helped you with …?					
Mitigating the risk of crisis					
- Trying to prevent a crisis	36 (12)	75 (28)	45 (26)	19 (9)	175 (19)
- Identifying patient’s deterioration	57 (20)	62 (23)	32 (18)	8 (4)	159 (17)
Managing caseload priorities	125 (43)	62 (23)	48 (27)	33 (16)	268 (28)
Nothing, it was not useful	73 (25)	72 (27)	50 (29)	145 (71)	340 (36)

The risk assessment was part of the feedback form delivered after an initial review of the presented cases (F1 in Table 2) with a completion rate of 92% (*n* = 935). One week after reviewing the patients flagged by the algorithm, CMHTs reassessed each case’s risk level. Their assessment of patient risk of crisis reduced in 17% of cases. Meanwhile, their perception of risk increased in 8% of cases. Clinicians rated the value of the risk predictions for mitigating the risk of crisis and for managing the caseload priority on the second feedback form (F2 in Table 2). The completion rate for F2 was 84% (*n* = 846) (see Table 1 for a detailed breakdown of the teams). Five months after the study started, semi-structured interviews were conducted to obtain additional insights into the algorithm’s implementation and the effect on decision-making in clinical practice (see the qualitative report in Supplementary Materials–Qualitative Evaluation).

#### Mitigating the risk of crisis

We evaluated the opportunity to mitigate the risk of a crisis using two questions that probed whether the algorithm helped identify patient deterioration and enabled a pre-emptive intervention to prevent a crisis. Predictions were rated useful in 64% (*n* = 602) of the presented cases overall and in more than 70% of cases in three of the four CMHTs. Only one CMHT (Team 4 in Table 2) reported no added value at a high percentage (71%; *n* = 145), with all other teams reporting percentages below 30%. Notably, CMHTs reported that the model was clinically valuable in terms of preventing a crisis in 19% (*n* = 175) of cases and in terms of identifying the deterioration of patient conditions in 17% (*n* = 159) of cases.

#### Managing the caseload

The value of our tool for managing caseload priorities was indirectly captured by analyzing whether risk predictions helped clinicians identify patient deterioration and decide which patients to contact. Managing caseload priorities is a complex task (especially in high-demand settings), and clinicians often rely on various parameters to prioritize caseloads, including prior knowledge about individual patients, subjective views about risks and diagnosis severity. Accordingly, we opted to capture the value of risk predictions using a general question that prompts clinicians to directly rate the value of the predictive tool for managing their caseload, with the responses indicating that the model output was used to manage caseload priorities in 28% (*n* = 268) of cases (see Table 2 for a detailed summary).

## Discussion

We have demonstrated the feasibility of predicting mental health crises by applying machine learning techniques to longitudinally collected EHR data, obtaining an AUROC of 0.797 for the general model. Despite the data availability concerns associated with the EHR (related to periods with no patient records), querying the prediction model continuously—that is, in a rolling window manner—produced a better performance than that obtained by the baseline models. The lack of records for more than 3 months resulted in a 7% drop in AUROC. Meanwhile, having no records about a patient for more than 6 months or 1 year contributed to drops of 13% and 20%, respectively. Unsurprisingly, having a longer data history improved the risk prediction performance for a given patient.

Among the machine learning models evaluated, XGBoost demonstrated the best overall performance. Nonetheless, in a few cases, there were only marginal or no significant improvements in comparison to other techniques (Extended Data Figs. 3 and 4). Training different models for each group of disorders to leverage the specificity of mental health disorders did not prove superior to the general model despite the differences in the performance of the general model for different disorders (Fig. 3c). No significant difference in performance was observed across different diagnostic groups, except for increased performance for organic disorders (likely due to their lower prevalence). We further expanded the subgroup analysis to assess the algorithm’s fairness. Among the common protected attributes (namely, gender, age, ethnic groups and disability), we observed a 5% increase in the AUROC for patients aged 65–74 years (likely a consequence of the considerably lower prevalence of this group) and a 7% lower AUROC for the ‘Black’ ethnic subgroup compared to the ‘White’ ethnic subgroup. We refrained from unpacking the potential causes of this disparate effect due to the complexity of known and unknown biases and factors that could not be controlled for (see Supplementary Materials–Fairness Analysis).

We evaluated whether a tool predicting and presenting risk of mental health crisis provides added value for clinical practice in terms of managing caseloads and mitigating the risk of crisis. On average, the CMHTs disagreed with only 7% of the model predictions, with the model outputs found to be clinically useful in 64% of individual cases. We did not successfully identify why considerably lower scores were observed in the responses from one of the four CMHTs, with neither the study process nor team and patient selection introducing any known bias. However, crucially, risk predictions were relevant to preventing crises in 19% of cases, to identifying the deterioration of a patient’s condition in 17% of cases and to managing caseload priorities in 28% (*n* = 268) of cases. Notably, the importance of the algorithm for identifying at-risk patients who would otherwise have been missed emerged from the semi-structured interviews conducted with the clinicians as part of the qualitative evaluation (see Supplementary Materials–Qualitative Evaluation). The relatively high percentage of cases (36%) in which predictions were not perceived as useful was substantially affected by the number of serious cases that were already being recognized and managed by the CMHTs. Nevertheless, the clinicians opted to receive the list of patients at the highest risk of experiencing a crisis even if doing so would mean including patients whom they were already monitoring. It is reasonable to expect that the requirements for the practical implementation would not be considerably different in other clinical settings. That is, broadening the prediction list to all patients registered in the hospital system would reduce the value of each prediction relative to clinician caseload, thus having little benefit.

Our study’s main limitation concerns the known and potentially unknown specificity of the single-center cohort. Given that EHRs are characterized by high dimensionality and heterogeneity, risk prediction algorithms suffer from overfitting the model to the data, which limits the generalizability of the results and undermines most predictive features. However, many data fields are expected to be routinely captured by typical mental health centers, even if they only register crisis emergencies, visits and hospitalizations. Based on this understanding, we selected only eight of the top 20 features derived solely from events related to crises, contacts and hospitalization (see the list in Supplementary Material–Crisis Prediction Model) and evaluated the corresponding model. The resulting AUROC was 0.781 (compared to 0.797 for the general model). Furthermore, we limited our algorithm’s applicability to patients with a history of relapse, a decision that was based on healthcare demand: patients prone to relapse require a considerable proportion of healthcare resources because they frequently need urgent and unplanned support, which engenders major challenges for optimizing healthcare resources. Thus, further research should probe the feasibility of developing an algorithm to detect first crises. Finally, although the clinicians reported that the prediction model helped to prevent a crisis in 19% of cases, this eventuality was not witnessed because it would have implied that the clinicians did not react to the predictions, which would have been ethically and legally unacceptable.

Machine learning techniques trained on historical patient records have demonstrated considerable potential to predict critical events in different medical domains (for example, circulatory failure, diabetes and cardiovascular disorders)^11–15^. In the mental health domain, prediction algorithms have typically focused on detecting individual propensity to die by suicide or develop psychosis, with no extant studies attempting to continuously detect important mental health events or those that would require readmission for urgent care or hospitalization. Nonetheless, several studies have considered predictions of unplanned hospital readmissions regardless of their underpinning reason^17,43–46^ and obtained AUROCs between 0.750 and 0.791 for predicting the risk of readmission within 30 days (similar to our results of 0.797 within 28 days). Although such algorithms can importantly benefit healthcare, their potential to improve caseload management or prevent unwanted health outcomes is limited by (1) the timing of queries (only at discharge rather than continuously) and (2) the nature of readmissions (not specific to any disorder in particular; as highlighted by the authors^46^ and the literature^47^, most such readmissions are not preventable). Running predictions continuously^13,14^ provides an updated risk score based on the latest available data, which typically contains the most predictive information, which is, in the case of mental health, crucial to improving healthcare management and outcomes.

The rising demand for mental healthcare is increasingly prompting hospitals to actively work on identifying novel methods of anticipating demand and better deploying their limited resources to improve patient outcomes and decrease long-term costs^9,48^. Evaluating technical feasibility and clinical value are critical steps before integrating prediction models into routine care models^32^. From this perspective, our study paves the way for better resource optimization in mental healthcare and enabling the long-awaited shift in the mental health paradigm from reactive care (delivered in the emergency room) to preventative care (delivered in the community).

## Methods

### Study design and setting

This study comprised two phases. The first phase involved a retrospective cohort study designed to build and evaluate a mental health crisis prediction model reliant on EHR data. The second phase implemented this model in clinical practice as part of a prospective cohort study to explore the added value it provides in the clinical context. Added value was defined as the extent to which the predictive algorithm could support clinicians in managing caseload priorities and mitigating the risk of crisis.

The retrospective and prospective studies were both conducted at Birmingham and Solihull Mental Health NHS Foundation Trust (BSMHFT). One of the largest mental health trusts in the UK, BSMHFT operates over 40 sites and serves a culturally and socially diverse population of over 1 million patients. The retrospective study used data collected between September 2012 and November 2018; the prospective study began on 26 November 2018 and ran until 12 May 2019.

### Ethical approval and consent

The Health Research Authority (HRA) approved the study. The HRA ensures that all NHS research governance requirements are met and that patients and public interests are protected. For the historical data used in the retrospective study, the need to obtain consent was waived on the basis of the use of anonymized data that cannot be linked to any individual patient. Furthermore, the consent form that had already been signed by patients upon joining the corresponding mental health service within the NHS included the potential purpose of using patient records for predictive risk analyses. Meanwhile, the participants in the prospective study were the healthcare staff members who consented to participation in the research and who had been trained in the use of the algorithm and its outputs in support of their clinical practice.

### Dataset

The dataset comprised anonymized clinical records extracted from a retrospective cohort of patients who had been admitted to BSMHFT. The data included demographic information, hospital contact details, referrals, diagnoses, hospitalizations, risk and well-being assessments and crisis events for all inpatients and outpatients. No exclusion criteria based on age or diagnosed disorder were applied, meaning that patient age ranged from 16 to 102 years and that a wide range of disorders was included. However, to include only patients with a history of relapse, patients who had no crisis episode in their records were excluded. This decision was made because detecting first crises and detecting relapse events correspond to different ground truth labels and different data. Furthermore, given that detecting relapse events can leverage information about the previous crisis, patients with only one crisis episode were excluded because their records were not suitable for the training and testing phases. Additionally, patients with three or fewer months of records in the system were excluded because their historical data were insufficient for the algorithm to learn from. For the remaining patients, predictions were queried and evaluated for the period after two crisis episodes and after having the first record at least 3 months before querying the model. This produced a total of 5,816,586 electronic records from 17,122 patients in the database used for this study. Supplementary Table 1 breaks down the number of records per type, and Supplementary Table 2 compares the representation of different ethnic groups and genders in the study cohort, the original hospital cohort and the Birmingham and Solihull area.

### Features and labels generation

With the exception of the static information, all EHR data included the associated date and time. The date and time refer to the moment when the specific event or assessment occurred—that is, the date and time that a patient was admitted to hospital or assigned a diagnosis. To prepare the data for the modeling task, each patient’s records were consolidated at a weekly level according to the date associated with the record. Following this process, we generated evenly spaced time series for each patient that spanned from the patient’s first interaction with the hospital to the study’s final week. The features and labels generated for each week were computed using the data with a date prior to that week. Static data susceptible to change over time (for example, marital status) were removed to mitigate the risk of retrospective leakage.

#### Label generation

To construct the binary prediction target, each patient-week was assigned a positive label whenever there was a relapse during the following 4 weeks (if the patient had not had a crisis during the current week) and a negative label otherwise. To assess the extent to which the model was sensitive to such a definition of the main label, we built 47 additional labels by varying three parameters:

- The number of stable weeks (without crisis) necessary to consider a crisis episode concluded: from 1 to 4 weeks.
- The prediction time window length (that is, the time window in which the algorithm assesses the risk of crisis): from 1 to 4 weeks.
- The number of weeks between the time of querying the algorithm and the start of the prediction time window: from 0 to 2 weeks.

#### Features generation

We extracted a total of 198 features from the ten data tables (Supplementary Table 5). Each data table was processed separately, and no imputation that could add noise to the data was performed. Feature extraction was performed according to six procedures:

- Static or semi-static features. Demographics data were represented as constant values attributed to each patient, with age treated as a special case that changed each year.
- Diagnosis features. Patients were assigned their latest valid diagnosed disorder or a ‘non-diagnosed’ label and then separated into diagnostic groups according to the latest valid diagnosed disorder at the last week of the training set to avoid leakage into the validation and test sets. Each diagnosed disorder was mapped to its corresponding first-level category according to the ICD-10 (ref. ^34^) code system. For instance, F200 paranoid schizophrenia disorder was mapped to the F2 Schizophrenia and Psychotic category. We shortened the names of the first-level ICD-10 categories for brevity and to improve figure layouts:
  - F0 Organic: organic, including symptomatic, mental disorders (ICD-10 codes F00–F09).
  - F1 Substance Misuse: mental and behavioral disorders caused by psychoactive substance use (ICD-10 codes F10–F19).
  - F2 Schizophrenia and Psychotic: schizophrenia and schizotypal and delusional disorders (ICD-10 codes F20–F29).
  - F3 Mood: mood (affective) disorders (ICD-10 codes F30–F39).
  - F4 Neurotic, Stress and Anxiety: neurotic, stress-related and somatoform disorders (ICD-10 codes F40–49).
  - F6 Personality and Behavior: disorders of adult personality and behavior (ICD-10 codes F60–69).
  - Other Diagnosis: any other disorder not contemplated by the previous categories (ICD-10 codes F50–59 and F70–99).
  - Not Diagnosed: no diagnosed disorder available in the EHR.
- EHR weekly aggregations. EHRs related to patient–hospital interactions were aggregated on a weekly basis for each patient. The resulting features constituted counts per type of interaction, one-hot encoded according to their categorization. If a specific type of event did not occur in a given week, a value of ‘0’ was assigned to the feature related to the corresponding type of event for the corresponding week.
- Time-elapsed features. At each patient-week, for each type of interaction and category, we constructed a feature that counted the number of weeks elapsed since the last occurrence of the corresponding event. If the patient had never experienced such an event type up to that point in time, NaN values were used.
- Last crisis episode descriptors. For each crisis episode, a set of descriptors summarizing the length and severity of the crisis episode was built. These descriptors were used to build features for the subsequent weeks until the next crisis occurred. If the patient had never had a crisis episode up to that point in time, NaN values were used.
- Status features. For specific EHRs that are characterized by the start–end date, features for the corresponding weeks were built by assigning their corresponding value (or category); otherwise, they were set to NaN.

In addition to EHR-based features, we also added the week number (of a year, 1–52) to account for seasonality effects. Given the cyclical nature of the feature, we encoded the information using the trigonometric transformations sine and cosine: sin($2\pi \frac{{week}}{{52}}$) and cos($2\pi \frac{{week}}{{52}}$).

### Crisis prediction modeling and evaluation

We defined the crisis prediction task as a binary classification problem to be performed on a weekly basis. For each week, the model predicts the risk of crisis onset during the upcoming 28 days. Applying a rolling window approach allows for a periodic update of the predicted risk by incorporating the newly available data (or the absence of it) at the beginning of each week. This approach is very common in settings where the predictions are used in real time and when the data are updated continuously, such as for predicting circulatory failure or sepsis intensive care units^13,14^.

We applied a time-based 80%/10%/10% training/validation/test split:

- Training data started in the first week of September 2012 and ended in the last week of December 2017.
- Validation data started in the first week of January 2018 and ended in the last week of June 2018.
- Test data started in the first week of July 2018 and ended in the third week of November 2018.

Performance evaluations were conducted on a weekly basis, and each week’s results were used to build CIs on the evaluated metrics. All reported results were computed using the test set if not otherwise indicated.

#### Machine learning classifiers

For our final models, we used XGBoost^49^, an implementation of gradient boosting machines (GBMs)^50^, and the best-performing algorithm. GBMs are algorithms that build a sequence of decision trees such that every new tree improves upon the performance of previous iterations. Given that XGBoost effectively handles missing data and is not sensitive to scaling factors, no imputation or scaling techniques were applied. For comparison, we also evaluated the performance of some state-of-the-art machine learning classifiers, including logistic regression, naive Bayes, random forest, isolation forest and neural networks (namely, multi-layer perceptron and long short-term memory recurrent neural networks, which have been used successfully in similar prediction studies based on EHR^51^). To ensure a fair comparison, standard scaling and imputation of missing values were performed for the classifiers that typically benefit from these procedures. We also performed 100 hyperparameter optimization trials for each classifier to identify the best hyperparameters. The search spaces are included in the Supplementary Materials (Supplementary Table 8).

#### Hyperparameter tuning and feature selection

To select the optimal hyperparameters for the trained models, we maximized AUROC based on the validation set using a Bayesian optimization technique. For this purpose, we used Hyperopt^52^, a sequential model-based optimization algorithm that performs Bayesian optimization via the Tree-structured Parzen Estimator^53^. This technique has a wide range of distributions available to accommodate most search spaces. Such flexibility makes the algorithm very powerful and appropriate for performing hyperparameter tuning on all of the classifiers used. The same methodology was used for feature selection. To that end, we grouped the features into categories based on the information gained and added a binary parameter assessing whether a particular feature should be selected (Supplementary Table 5).

#### Model interpretation

We used SHAP values to measure the contribution that each feature made to the main model^42^. This technique is based on the Shapley value from game theory, which quantifies the individual contributions of all the participants of a game to the outcome and represents the state-of-the-art approach to interpreting machine learning models. SHAP values were computed using the Python package shap, version 0.35.0, and the TreeExplainer algorithm, an additive feature attribution method that satisfies the properties of local accuracy, consistency and allowance for missing data^54^. Feature attributions are computed for every particular prediction, assigning each feature an importance score that considers interactions with the remaining features. The resulting SHAP values provide an overview of the feature’s contribution based on its value and allow for both local and global interpretation. All SHAP values were computed from the test set.

To further evaluate the stability of the model and its interpretation, we conducted an experiment in which we generated 100 different samples by randomly selecting 40% of the patients per sample. We trained a model for each of the 100 samples and computed the SHAP values for the whole test set. The consistency of the most important predictors was evaluated through the cosine similarity between the SHAP values of the top 20 features of the final model and the models trained on each of the 100 samples. The results (presented in Supplementary Materials–Stability of Most Predictive Features) were consistent with the analysis of the general model.

### Statistical methods

If not otherwise indicated, all reported metrics in text, tables and figures refer to the performance evaluation on the test set. CIs for the reported performance metrics were computed using *n* = 25 temporal splits. Statistical analysis for model comparison was conducted based on the AUROC and its equivalence to the Mann–Whitney *U*-statistic and following the theory surrounding using generalized *U*-statistics to compare correlated ROC curves^55^. The two-stage step-up method of Benjamini, Krieger and Yekutieli^56^ was used to correct the *P* values of the multiple tests performed. For figures showing curves (Figs. 3a,b and 4c–h, Extended Data Fig. 6c and Supplementary Fig. 1), solid lines and shaded areas correspond to the means and standard deviations of the performance metrics across the temporal splits in the test set. For figures featuring point plots (Fig. 3d–f and Extended Data Fig. 8a–f), center points and vertical bars correspond to the means and 95% CIs across the temporal splits in the test set. For box plot figures (Fig. 3c and Extended Data Fig. 7a–c), the solid line corresponds to the median value; the box limits correspond to the first (left limit) and third (right limit) quartiles; the whiskers denote the rest of the distribution range from Q1–1.5 (Q3–Q1) (left whisker) to Q3 + 1.5 (Q3–Q1) (right whisker); and the points displayed correspond to the outliers.

We evaluated the calibration of our proposed model and the model for each diagnosis, meaning that we compared the PRS of the model to the observed risk aggregating the observed labels. To calibrate the risk scores, we fitted an isotonic regression model^38^ to the validation set’s predictions and transformed the test set’s predictions. Consequently, the transformation applied to the PRS preserves the rank and minimizes the deviation between the actual target variable and the final PRS. We used 25 evenly spaced bins on the PRS to generate the calibration curve in Extended Data Fig. 6a,b

### Clinical evaluation

#### Participants

A total of 60 clinicians from four CMHTs participated in the study. Four were doctors, two were occupational therapists, two were duty workers, one was a social worker and 51 were nurses, including clinical leads and team managers (see Table 1 for an overview of the CMHTs). Each team had at least two coordinators who served as the first contact point for their team and who were responsible for assigning individual cases to the participating clinical staff. The four CMHTs reviewed crisis predictions from a total number of 1,011 cases in a prospective manner as part of their regular clinical practice. Although the initial plan was to include 1,200 cases, 189 cases were discarded from the analysis due to an internal technical error. Crucially, this error did not affect the study results beyond slightly reducing the sample size.

#### Data collection

The general model, using the most recent available data, was applied on a biweekly basis to generate the PRS for all patients. Patients were ranked, and each CMHT received a list of the 25 patients (belonging to their caseload) at greatest risk of crisis. The tool used by the participants contained a list of patient names and identifiers, risk scores and relevant clinical and demographic information (Supplementary Table 10).

Upon reviewing the list of patients, the CMHTs completed the F1 feedback form, which asked them to:

- Provide their assessment of each patient’s crisis risk level and indicate agreement or disagreement with the algorithm-based prediction.
- Specify their intended action in response to each prediction.

One week after the initial review, the CMHTs completed the F2 feedback form, which asked them to:

- Provide each patient’s crisis risk level, based on further assessment, and indicate whether the tool had influenced them to change their previous assessment.
- Indicate whether the algorithm-based predictions contributed valuably to managing caseload priority or mitigating the risk of crisis (due to early identification of symptomatic deterioration, enabling them to provide support or attempt to prevent a crisis).

Finally, five staff members (three community psychiatric nurses, one psychiatrist and one team manager) were individually interviewed and responded to a set of open-ended questions that concerned the added value of the crisis prediction model, its implementation and the facilitators and barriers to its use in practice. The interviews were conducted 5 months after the start of the study to sufficiently expose participants to the crisis prediction algorithm (see Supplementary Materials–Qualitative Evaluation for the interview reports).

### Reporting Summary

Further information on research design is available in the Nature Research Reporting Summary linked to this article.

## Online content

Any methods, additional references, Nature Research reporting summaries, source data, extended data, supplementary information, acknowledgements, peer review information; details of author contributions and competing interests; and statements of data and code availability are available at 10.1038/s41591-022-01811-5.

## Supplementary information

Supplementary Information Supplementary Data Summary, Fairness Analysis, Supplementary Results, Supplementary Model Interpretation, Stability of Most Predictive Features Analysis, Qualitative Evaluation of Prospective Study, Supplementary Tables 1–10 and Supplementary Figs. 1–4

Reporting Summary

## Data Availability

EHRs that support this study’s findings contain highly sensitive information about vulnerable populations and, therefore, cannot be made publicly available. Any request to access the data will need to be reviewed and approved by the Birmingham and Solihull Mental Health NHS Foundation Trustʼs Information Governance Committee.

## Code availability

The code that supports this study’s findings was tailored to the data from the hospital’s database and its structure. Therefore, the code has little use without access to the data and, as such, has not been made publicly available. All data processing and modeling were conducted on Python 3.6.7 using standard libraries that are publicly available: pandas, numpy, scipy, scikit-learn, xgboost, keras, tensorflow, matplotlib, seaborn, pymssql, jupyter shap and hyperopt.
