# Supplementary material for: Machine learning model to predict mental health crises from electronic health records
Source: Nat Med. 2022 May 16;28(6):1240–8. doi: 10.1038/s41591-022-01811-5 (PMC9205775; doi:10.1038/s41591-022-01811-5)
Supplement: Supplementary file 1 — Supplementary Data Summary, Fairness Analysis, Supplementary Results, Supplementary Model Interpretation, Stability of Most Predictive Features Analysis, Qualitative Evaluation of Prospective Study, Supplementary Tables 1–10 and Supplementary Figs. 1–4 [file 41591_2022_1811_MOESM1_ESM.pdf]

nature portfolio

https://doi.org/10.1038/s41591-022-01811-5

Supplementary information

Machine learning model to predict mental health crises from electronic health records

In the format provided by the authors and unedited

Supplementary Information

Data summary

Supplementary Table 1 shows statistics of different records and variables that were used to extract the features. Expectedly, most of the records belong to Contacts and Crisis Events followed by Hospitalisations. Contact records represent visits or calls that a patient (or a corresponding individual, such as a family member or their caregiver) had with the hospital. Crisis Events were recorded when patients accessed any of the urgent care pathways of the Trust. Hospitalisation events tracked each day when a patient stayed at the hospital over night. Referrals contain information about the team to which a patient was allocated to, the source of the referral and the time period that a patient has been referred to that team. Risk and wellbeing assessments were performed by the Trust and recorded in the EHRs. Crisis plans contain information about each time a new action plan was introduced when a patient had a crisis even was created or updated. Mental health Act refers to the information about the legal relationship between a patient and the hospital. Diagnosis records contain one or multiple diagnosed disorders for each patient. Demographics is the table with the latest information about the patient's general characteristics and demographics.

Supplementary Table 1 Number of records and variables per data source.

Table	Num records (%)	Num variables (%)
Contacts	2,386,631 (42.5)	5 (6.8)
Crisis Events	1,522,548 (26.2)	4 (5.4)
Hospitalisations	997,331 (17.1)	4 (5.4)
Referrals	269,308 (4.6)	6 (8.1)
Risk Assessments	262,489 (4.5)	12 (16.2)
Wellbeing Assessments	128,121 (2.2)	7 (9.5)
Crisis Plans	107,197 (1.8)	3 (4.1)
Mental Health Act	84,991 (1.4)	5 (6.8)
Diagnosis	37,534 (0.6)	16 (21.6)
Demographics	20,436 (0.4)	6 (8.1)
Total	5,816,586 (100)	74 (100)

Fairness analysis

Background

Providing mental health crisis predictions in clinical settings entails various decisions that affect human lives. Evaluating the extent of a systematic discrimination against individuals based on certain attributes is of a paramount importance before deploying predictive algorithms. With the lack of a solid consensus in formal definition of fairness, we adopted the process of assessing algorithmic biases based on common protected variables. Although any variable that may raise individual or public concerns can be categorised as protected[1], we focus on the most common ones, namely gender, ethnicity, and disability. We recognise that in the context of mental disorders, age, disorder type and socioeconomic status also represent highly important variables. The prediction performance was analysed for different age groups and disorder types as part of the main performance analysis (see Results section and Performance for different age groups section in Supplementary Information), whereas socioeconomic indicators were not available in EHRs used in this study.

Representation of different gender and ethnic Groups

Gender and ethnic distribution in the EHRs used in this study prior to applying the inclusion criteria (i.e. in the hospital cohort) reflect the population of Birmingham & Solihull area (B&S), that is a geographic area covered by the hospital (i.e. NHS Trust). The proportion of females in the hospital cohort is comparable to the relative proportion in the B&S area, 53.5% and 50.9% respectively. The proportion of patients with ethnic characteristic "White" and "Black" in the hospital cohort (73.5% and 5.8% respectively) were also comparable to the B&S population (71.8% and 5.7% respectively). The proportion of patients with ethnic characteristic "Asian" and "Mixed" in the hospital cohort (14.0% and 6.7% respectively) deviated from the statistics in the corresponding geographical area – the former group was slightly underrepresented while the latter was slightly overrepresented when compared to the B&S residents (17.7% and 3.3% respectively).

After applying the inclusion criteria, the percentage of females in the study cohort (used for the data analysis) decreased to 48.6%, which is closer to the population in the B&S area (50.9%) than in the original hospital cohort. "Black" and "Mixed"

groups in the study cohort (8.9% and 7.02% respectively) were overrepresented when compared to the B&S residents (5.7% and 3.3% respectively). These ethnic groups typically show a greater prevalence of Psychotic and Mood disorders with respect to the general population[2–4]. Overrepresentation of these groups within the study cohort consequently resulted in a slight decrease in the relative percentage of the patients with ethnic characteristic "White" (66.1%), and in a slight increase in the relative percentage of Asian patients (with 14.8% who remained underrepresented as compared to 17.7% in B&S area). Moreover, it is possible that some of the wider determinants of health may affect access to some health services, potentially leading to a greater use of hospital services, which would also lead to over-representation in hospital services. However, such an analysis is out of scope for this paper.

Supplementary Table 2 Representation of different gender and ethnic groups in the study cohort, the hospital cohort and the Birmingham & Solihull population.

	**Study cohort**	**Hospital cohort**	**Birmingham & Solihull area**
Gender			
Male	51.4%	46.5%	49.1%
Female	48.6%	53.5%	50.9%
Ethnic group			
White	66.1%	73.5%	71.8%
Asian	14.8%	14.0%	17.7%
Black	8.9%	5.8%	5.7%
Mixed	7.02%	6.7%	3.3%

Notwithstanding the minor discrepancies, all gender and ethnic groups in the B&S area have a reasonable representation in our EHRs (both in the study cohort and in the original hospital cohort) in comparable ratios. Upon applying the inclusion criteria, the relative representation of patients with ethnic characteristics "White" and "Asian" slightly decreased whereas the percentage of those with ethnic characteristic "Black" and "Mixed" increased, which is likely due to a higher prevalence of Psychotic and Mood disorders in Black and Mixed populations (Supplementary Table 2 shows the summary). Mental health disorders frequently imply disability, therefore comparing the representation of patients with disability in our cohorts with the general population in B&S area would not be meaningful.

Algorithm fairness analysis

To evaluate algorithmic fairness, we analyse the algorithm outputs and performance across the protected attributes of gender, ethnicity and disability. Firstly, we compare the aggregated rates at which the algorithm a) flagged patients at risk (both correctly or incorrectly), and b) correctly detected which patients were actually at risk. Secondly, we delve into the algorithm performance by analysing disparate impact.

The algorithm fairness literature does not provide a clear-cut threshold for disparate impact which is considered as discrimination, rather they typically refer to the 80% rule inspired by the US Equal Employment Opportunity Commission 80:100[5,6]. This rule defines that the ratio between the percentage of individuals that have a specific protected attribute assigned the positive decision outcome and the percentage of individuals not having that value also assigned the positive outcome should not be less than. In our fairness analysis, we did not observe major discrepancies in the algorithm performance among patients with different protected attributes exceeding this threshold thus indicating positive or negative discrimination. A very few exceptions are discussed in the following section. However, we arbitrarily established a threshold of 5% to highlight a difference in the algorithm output among people with different protected attributes.

Discrimination of different patient groups

We study disparate impact in the number of crises predicted by the algorithm in each group (i.e. protected attribute) by juxtaposing three different variables, (1) number of crises occurred in each group expressed as a percentage of the total number of crises in the cohort, (2) percentage of patients flagged by the algorithm to be at risk of a crisis, per each group, (3) percentage of correctly identified cases at risk, per each group (denoted as “Crises occurred”, “Crisis flagged", and “Crisis detected” respectively in Supplementary Table 3).

The proportions of patients per each protected attribute flagged by the algorithm follow (within $\pm$5%) the proportion of crises that occurred in practice, with a few exceptions. The patients whose disability status was unknown were flagged by the

algorithm 9% less frequently than the baseline, whereas patients with disability were flagged with an 8% rate above the baseline rate. The lack of ethnicity labels (“Not Known” in Supplementary Table 3) resulted in a 25% higher rate of being flagged as patients at risk compared to the baseline, although they only represent the 2.3% of the occurred crises. Even though this theoretically exceeds the 80% rule and it can be interpreted as positive discrimination, we do not consider that the algorithm is treating a specific group of patients in an unfair manner given that the protected variable is unknown.

The proportion of correctly identified crises was also within ±5% of the baseline for most of the protected attributes. However, for the patients with ethnic characteristic "Black", the algorithm detected 13% fewer crises than the baseline. For this ethnic group, the algorithm was flagging the risk of crisis at a representative amount (*i.e.*, only 1% above) which suggests a higher false positive rate. The other exception occurred in patients with disability (9% above baseline), which was consistent with the fact that there was a higher rate of the flagged patients in this group (8%).

Disparate impact analysis

Following the standard literature[7], we selected three metrics to assess disparate impact across patients with different protected attributes, namely:

- False negative rate (FNR) represents the percentage of positive cases incorrectly labeled as negative. In other words, for the obtained rate of crises that were not detected, this parameter reflect disparate impact of the algorithm (FNR discrepancy).
- Predictive positive value (PPV) indicates the percentage of true positives among all the positive predictions (also referred to as precision).
- AUROC measures how well the algorithm separates positive and negative labels within each group.

The three selected metrics are reported in Supplementary Table 4. No disparate impact was witnessed between the two gender groups (all differences in the corresponding rations are below 5%). Positive disparate impact was identified for patients with disability, resulting in a PPV 9% above and a FNR 17% below the rates for patients without disability respectively. In addition, there was a negative difference in AUROC (4%) and a higher percentage of crises flagged and detected for patients with a disability, which together with a discrepancy in PPV and FNR indicates the the algorithm overestimates the crisis risk for this protected attribute, although not to a large extent. The highest negative disparate impact was observed for patients with ethnic characteristic "Black", with a PPV 17% below and a FNR 22% above patients with ethnic characteristic "White". Furthermore, the AUROC for patients with Black ethnicity was 7% below the AUROC for patients with White ethnicity. As the percentage of crises flagged by the algorithm was highly comparable to the one recorded in practice, the disparity analysis suggests that detection of crises for patients with Black ethnicity is more challenging than for the other ethnic groups. Delving into the root causes of this disparate impact represents a complex endeavour that is out of the scope of this paper. This is due to multiple known and unknown factors (such as differences in the underlying wider determinants of health within the B&S geography that might affect risk factors, service attendance, adherence to therapy, etc, amongst different ethnic populations) that may have caused either discrepancies in EHRs, or resulted in a different phenomenology in detecting mental health crises. For this, we refrain from further analysis and from making firm conclusions.

Supplementary Table 3 Percentage of crisis episodes occurred, flagged by the algorithm and correctly detected among the subgroups. Ratio, in brackets, was computed by dividing the number of crises that were flagged / detected by the number of crises that occurred in practice.

	Crises occurred %	Crises flagged % (ratio)	Crises detected % (ratio)
Gender			
Male	50.8%	51.1% (1.01)	51.6% (1.02)
Female	49.2%	48.8% (0.99)	48.4% (0.98)
Ethnic group			
White	68.1%	67.7% (1.00)	70.1% (1.03)
Asian	14.4%	14.3% (0.99)	13.5% (0.94)
Black	9.0%	9.1% (1.01)	7.9% (0.87)
Mixed	6.2%	5.9% (0.96)	6.4% (1.03)
Not known	2.3%	2.9% (1.25)	2.1% (0.92)
People with Disability			
Disabled	35.2%	37.9% (1.08)	38.3% (1.09)
Not disabled	27.4%	28.2% (1.03)	26.2% (0.96)
Not known	37.4%	34.0% (0.91)	35.5% (0.95)

Supplementary Table 4 Positive predictive value, false negative rate, and AUROC per subgroup. The ratios, in brackets, were computed by dividing the value of each metric by the value corresponding to the baseline group.

	Predicted Positive Value (ratio)	False Negative Rate (ratio)	AUROC (ratio)
Gender			
Male (base)	0.110 (1.00)	0.418 (1.00)	0.802 (1.00)
Female	0.108 (0.98)	0.436 (1.04)	0.793 (0.99)
Ethnic group			
White (base)	0.113 (1.00)	0.409 (1.00)	0.805 (1.00)
Asian	0.103 (0.91)	0.464 (1.13)	0.789 (0.98)
Black	0.094 (0.83)	0.500 (1.22)	0.747 (0.93)
Mixed	0.118 (1.04)	0.407 (0.99)	0.799 (0.99)
Not known	0.081 (0.71)	0.474 (1.16)	0.770 (0.96)
People with Disability			
Disabled	0.111 (1.09)	0.376 (0.83)	0.778 (0.96)
Not disabled (base)	0.102 (1.00)	0.452 (1.00)	0.807 (1.00)
Not known	0.114 (1.12)	0.456 (1.01)	0.798 (0.99)

Crisis prediction model

Feature inclusion

Supplementary Table 5 presents each feature that was extracted, alongside its source table and its inclusion in the final model. In total, 198 features were computed, with 104 that were selected for the final model.

Supplementary Table 5. Complete list of features

Feature name	Source table	Included in final model
Contact not attended without follow-up	Contacts	Yes
Contact within last 24 weeks	Contacts	Yes
Contact within last 4 weeks	Contacts	Yes
Never contact event	Contacts	Yes
Weeks since last contact	Contacts	Yes
Weeks since last missed appointment	Contacts	Yes
Weeks since last contact with carer	Contacts	Yes
Weeks since last face-to-face contact	Contacts	Yes
Weeks since last group contact	Contacts	Yes

Weeks since last contact (other)	Contacts	Yes
Weeks since last revision contact	Contacts	Yes
Weeks since last contact by phone	Contacts	Yes
Weeks since last unplanned contact	Contacts	Yes
Contact	Contacts	No
Not attended contact	Contacts	No
Contact with carer	Contacts	No
Face-to-face contact	Contacts	No
Group contact	Contacts	No
Other type of contact	Contacts	No
Revision contact	Contacts	No
Contact by phone	Contacts	No
Unplanned contact	Contacts	No
Number of contacts during week	Contacts	No
Crisis within the last 4 weeks	Crisis Events	Yes
Crisis within the last 8 weeks	Crisis Events	Yes
Maximum severity during last crisis	Crisis Events	Yes
Never crisis event	Crisis Events	Yes
Number of crisis episodes	Crisis Events	Yes
Number of crisis events during last crisis	Crisis Events	Yes
Number of days in crisis during last crisis	Crisis Events	Yes
Weeks since last crisis	Crisis Events	Yes
Crisis allocation type bed management day	Crisis Events	No
Crisis allocation type pdu day	Crisis Events	No
Crisis allocation type po day	Crisis Events	No
Crisis allocation type contact	Crisis Events	No
Crisis allocation type IP bed	Crisis Events	No
Crisis allocation type ooa	Crisis Events	No
Crisis allocation type rnc	Crisis Events	No
Crisis allocation type st	Crisis Events	No
Crisis type bm	Crisis Events	No
Crisis type ip	Crisis Events	No
Crisis type ooa	Crisis Events	No
Crisis type tr	Crisis Events	No
Crisis event	Crisis Events	No
No crisis event in at least one day	Crisis Events	No
Number of crisis events during week	Crisis Events	No
Maximum number of crisis events in a week	Crisis Events	No
Number of crisis events	Crisis Events	No
Number of crisis episodes during week	Crisis Events	No
Number of crisis 2 week episodes during week	Crisis Events	No
Maximum severity during week	Crisis Events	No
Crisis plan up to date	Crisis Plans	Yes
Current age	Demographics	Yes
Older than 65	Demographics	Yes
Gender female	Demographics	Yes
Number of years since first visit	Demographics	Yes
Number of months since first visit	Demographics	No
Ethnic group Asian	Demographics	No
Ethnic group Black	Demographics	No
Ethnic group Mixed	Demographics	No
Ethnic group not known	Demographics	No
Ethnic group other	Demographics	No
Ethnic group White	Demographics	No
Gender male	Demographics	No

Marital status co habitee	Demographics	No
Marital status divorced	Demographics	No
Marital status married	Demographics	No
Marital status not asked	Demographics	No
Marital status not disclosed	Demographics	No
Marital status not recorded	Demographics	No
Marital status other unknown	Demographics	No
Marital status separated	Demographics	No
Marital status single	Demographics	No
Marital status unknown	Demographics	No
Marital status widowed	Demographics	No
F0 Organic including symptomatic mental disorders	Diagnosis	Yes
F1 Mental and behavioural disorders due to psychoactive substance use	Diagnosis	Yes
F2 Schizophrenia schizotypal and delusional disorders	Diagnosis	Yes
F3 Mood affective disorders	Diagnosis	Yes
F4 Neurotic stress related and somatoform disorders	Diagnosis	Yes
F6 Disorders of adult personality and behaviour	Diagnosis	Yes
Not diagnosed	Diagnosis	Yes
Other diagnosis	Diagnosis	Yes
Dual diagnosis	Diagnosis	Yes
Never diagnosed	Diagnosis	Yes
Hospitalized with level of observation 1 during last crisis	Hospitalizations	Yes
Number of days hospitalized with level of observation 1 during last crisis	Hospitalizations	Yes
Hospitalized with level of observation 2 during last crisis	Hospitalizations	Yes
Number of days hospitalized with level of observation 2 during last crisis	Hospitalizations	Yes
Hospitalized with level of observation 3 during last crisis	Hospitalizations	Yes
Number of days hospitalized with level of observation 3 during last crisis	Hospitalizations	Yes
Hospitalized with level of observation 4 during last crisis	Hospitalizations	Yes
Number of days hospitalized with level of observation 4 during last crisis	Hospitalizations	Yes
Maximum level of observation hospitalized during last crisis	Hospitalizations	Yes
Maximum length of stay during last crisis	Hospitalizations	Yes
Never hospitalized	Hospitalizations	Yes
Number of days hospitalized during last crisis	Hospitalizations	Yes
Number of days in leave while hospitalized during last crisis	Hospitalizations	Yes
Never needed MHA	Mental Health Act	Yes
CTO status active	Mental Health Act	No
CTO status not applicable	Mental Health Act	No
CTO status recalled	Mental Health Act	No
Under MHA section code	Mental Health Act	No
Week of the year	No source table	Yes
Cosine week of the year	No source table	Yes
Sine week of the year	No source table	Yes
Year	No source table	No
Never referral	Referrals	Yes
Weeks since last referral	Referrals	Yes
Weeks since last completed treatment discharge	Referrals	Yes
Weeks since last discharge for declined treatment	Referrals	Yes
Weeks since last discharge for missed appointment	Referrals	Yes
Weks since last internal discharge	Referrals	Yes
Weeks since last discharge for no mental health	Referrals	Yes
Weeks since last discharge for not suitable treatment	Referrals	Yes
Weeks since last discharge other	Referrals	Yes
Weeks since last discharge for security	Referrals	Yes
Weeks since last referral from acute services	Referrals	Yes
Weeks since last referral from ambulance	Referrals	Yes

Weeks since last referral from carer	Referrals	Yes
Weeks since last referral from community	Referrals	Yes
Weeks since last referral from gp	Referrals	Yes
Weeks since last referral from internal services	Referrals	Yes
Weeks since last referral from local authority	Referrals	Yes
Weeks since last referral from mental health services	Referrals	Yes
Weeks since last referral from other agency	Referrals	Yes
Weeks since last referral from primary care	Referrals	Yes
Weeks since last self referral	Referrals	Yes
Referral	Referrals	No
Completed treatment discharge	Referrals	No
Discharge for declined treatment	Referrals	No
Discharge for missed appointment	Referrals	No
Internal discharge	Referrals	No
Discharge for no mental health	Referrals	No
Discharge for not suitable treatment	Referrals	No
Discharge other	Referrals	No
Discharge for security	Referrals	No
Referral from acute services	Referrals	No
Referral from ambulance	Referrals	No
Referral from carer	Referrals	No
Referral from community	Referrals	No
Referral from gp	Referrals	No
Referral from internal services	Referrals	No
Referral from local authority	Referrals	No
Referral from mental health services	Referrals	No
Referral from other agency	Referrals	No
Referral from primary care	Referrals	No
Self referral	Referrals	No
Completed treatment discharge state	Referrals	No
Discharge for declined treatment state	Referrals	No
Discharge for missed appointment state	Referrals	No
Internal discharge state	Referrals	No
Discharge for no mental health state	Referrals	No
Discharge for not suitable treatment state	Referrals	No
Discharge other state	Referrals	No
Discharge for security state	Referrals	No
Referral from acute services state	Referrals	No
Referral from ambulance state	Referrals	No
Referral from carer state	Referrals	No
Referral from community state	Referrals	No
Referral from gp state	Referrals	No
Referral from internal services state	Referrals	No
Referral from local authority state	Referrals	No
Referral from mental health services state	Referrals	No
Referral from other agency state	Referrals	No
Referral from primary care state	Referrals	No
Self referral state	Referrals	No
Number of referrals during week	Referrals	No
Never risk assessment	Risk Assessments	Yes
Risk assessment up to date	Risk Assessments	Yes
Risk of forensic care	Risk Assessments	Yes
Risk of abusing medicine	Risk Assessments	Yes
Risk of absconding	Risk Assessments	Yes
Risk of accident	Risk Assessments	Yes

Risk of harm to others	Risk Assessments	Yes
Risk of violence	Risk Assessments	Yes
Risk of self harm	Risk Assessments	Yes
Risk of self neglect	Risk Assessments	Yes
Risk of substance misuse	Risk Assessments	Yes
Risk of suicide	Risk Assessments	Yes
Risk to children	Risk Assessments	Yes
Weeks since last risk of forensic care identified	Risk Assessments	Yes
Weeks since last risk of abusing medicine identified	Risk Assessments	Yes
Weeks since last risk of absconding identified	Risk Assessments	Yes
Weeks since last risk of accident identified	Risk Assessments	Yes
Weeks since last risk of harm to others identified	Risk Assessments	Yes
Weeks since last risk of violence identified	Risk Assessments	Yes
Weeks since last risk of self harm identified	Risk Assessments	Yes
Weeks since last risk of self neglect identified	Risk Assessments	Yes
Weeks since last risk of substance misuse identified	Risk Assessments	Yes
Weeks since last risk of suicide identified	Risk Assessments	Yes
Weeks since last risk to children identified	Risk Assessments	Yes
Never wellbeing assessment	Wellbeing Assessments	Yes
Wellbeing assessment emotional score	Wellbeing Assessments	Yes
Wellbeing assessment four factor total	Wellbeing Assessments	Yes
Wellbeing assessment personal score	Wellbeing Assessments	Yes
Wellbeing assessment severe disturbance score	Wellbeing Assessments	Yes
Wellbeing assessment social score	Wellbeing Assessments	Yes

Out of the 20 most predictive features in the general model, we selected the list of 8 features derived solely from records corresponding to crisis, contacts, diagnosis, demographics and hospitalisation events, including:

- Weeks since last crisis
- Never hospitalized
- Number of crisis episodes
- Number of years since first visit
- Weeks since last missed appointment
- Age
- Not diagnosed
- F6 Disorders of adult personality and behaviour

To the best of our knowledge, these records are typically available across a wide range of mental health hospitals. We extracted the selected features and evaluated the accuracy of the corresponding model to discuss (although speculatively) the generalisability of our results.

Evaluation of different machine learning techniques

We tested a wide range of classifiers, presented in Supplementary Table 6. XGBoost demonstrated the highest accuracy across different metrics, followed by Neural Networks, Random Forest and Logistic Regression. Additionally, we tested an unsupervised anomaly detection algorithm (Isolation Forest), which performed poorly in comparison to the machine learning techniques.

Supplementary Table 6 Evaluation of multiple Machine Learning models to predict the risk of crisis onset during the following 28 days. Values in bold denote the model with the highest performance.

Model	AUROC (std)	AP (std)
Clinical baseline	0.736 (0.010)	0.092 (0.006)
Diagnosis baseline	0.746 (0.011)	0.092 (0.006)
XGBoost	**0.797 (0.012)**	**0.159 (0.014)**
Logistic Regression	0.788 (0.010)	0.140 (0.009)
Random Forest	0.788 (0.012)	0.143 (0.013)
Decision Tree	0.776 (0.011)	0.118 (0.007)
Naive Bayes	0.751 (0.011)	0.108 (0.009)
SGD (modified huber)	0.785 (0.010)	0.134 (0.008)
Feed Forward Neural Network	0.790 (0.011)	0.145 (0.010)
LSTM	0.775 (0.015)	0.148 (0.013)
Isolation Forest	0.542 (0.009)	0.034 (0.003)

Supplementary Table 7 Statistical significance analysis comparing the AUROC achieved by XGBoost with a range of different Machine Learning techniques

Model	Z-statistic	P-value before correction	P-value after correction
Clinical baseline	19.10	2.32e-81	2.19e-80
Diagnosis baseline	16.38	2.76e-60	1.30e-59
Logistic Regression	3.19	0.0014	0.0018
Random Forest	3.17	0.0015	0.0018
Decision Tree	7.00	2.63e-12	4.98e-12
Naive Bayes	14.72	4.92e-49	1.55e-48
SGD (modified huber)	3.39	0.00069	0.0011
Neural Network	2.39	0.017	0.018
LSTM	7.10	1.23e-12	2.90e-12
Isolation Forest	77.13	0	0

Supplementary Table 8. Hyperparameter space explored for each model.

Hyperparameter	Range	Sampling	Final value
XGBoost			
Max depth tree	{3,...,16}	Uniform	8
Min child weigth	{80,...,150}	Uniform	103
Learning rate	[0.001,0.05]	Loguniform	0.011
Gamma	[80,170]	Uniform	120.65
Alpha regularisation	[170,300]	Uniform	226.52
Lambda regularisation	[0.1,10]	Uniform	2.85
Column subsampling	[0.5,1]	Uniform	0.759
Row subsampling	[0.6,0.9]	Uniform	0.778
Preprocessor	{None, PowerScale, Standard, Robust, MinMax}	Choice	None
Logistic Regression			
Solver	{liblinear, lbfgs, saga}	Choice	liblinear
Penalty	{l1, l2, elasticnet}	Choice	l2
C	[0.001, 100]	Uniform	51.96
Max iterations	{50, 55,..., 1000}	Uniform	245
L1 ratio	[0,1]	Uniform	-
Preprocessor	{None,PowerScale, Standard, Robust, MinMax}	Choice	PowerScale
Decision Tree			
Criteria	{gini, entropy}	Choice	entropy
Min samples split	{0, 100, ..., 50000}	Uniform	30000

Min samples leaf	{0, 100, ..., 50000}	Uniform	15800
Min weight sample leaf	[0,0.4]	Uniform	0.00730
Max features	[None, log2, sqrt]	Choice	None
Min impurity decrease	[$1e^{-7}$,1]	LogUniform	$3.12e^{-7}$
Preprocessor	{None, PowerScale, Standard, Robust, MinMax}	Choice	Standard
Random Forest			
Num estimators	{100, 150, ..., 400}	Uniform	250
Criterion	{gini, entropy}	Choice	gini
Max depth	{2,...16}	Uniform	9
Max features	{log2, sqrt, 0.2, 0.4, 0.6, 0.8}	Choice	0.4
Max sample	[0.1, 0.9]	Uniform	0.405
Warm start	{True, False}	Choice	True
Preprocessor	{None,PowerScale, Standard, Robust, MinMax}	Choice	PowerScale
Naive Bayes			
Variance smoothing	[$1e^{-9}$, 1000]	Loguniform	737.14
Preprocessor	{None, PowerScale, Standard, Robust, MinMax}	Choice	Standard
SGD (modified huber)			
Penalty	{l1, l2, elasticnet}	Choice	l1
Alpha	[$1e^{-6}$,1]	Loguniform	0.00329
L1 ratio	[0,1]	Uniform	-
Max iterations	{100, 200, ..., 10000}	Uniform	5600
Preprocessor	{None,PowerScale, Standard, Robust, MinMax}	Choice	PowerScale
Multi layer Perceptron			
Learning rate	[$1e^{-7}$,0.1]	Loguniform	$2.09e^{-5}$
Number layers	{1,...,10}	Uniform	6
Layer size	{20,40,...200}	Uniform	160
Batch size	{128,256,512,1024}	Choice	512
Epochs	{10, 15, ...50}	Uniform	35
Batch normalisation	{True, False}	Choice	True
Dropout rate	[0.05,0.7]	Uniform	0.39
Preprocessor	{None,PowerScale, Standard, Robust, MinMax}	Choice	PowerScale
LSTM			
Learning rate	[$1e^{-7}$,$1e^{-4}$]	Loguniform	$3.70e^{-6}$
Number dense layers	{1,...,16}	Uniform	2
Dense layer size	{20,40,...1024}	Uniform	440
LSTM layer size	{20,40,...1020}	Uniform	700
Batch size	{128,256,512,1024, 2048}	Choice	1024
Epochs	{10, 15, ...50}	Uniform	15
Batch normalisation	{True, False}	Choice	False
Dropout rate	[0.05,0.7]	Uniform	0.20
Preprocessor	{None,PowerScale, Standard, Robust, MinMax}	Choice	PowerScale
Isolation Forest			
Number estimators	{50,70,...,500}	Uniform	60
Max sample	{'auto',0.1,0.2,0.3,0.4,0.5,0.6,0.7,0.8,0.9}	Choice	'auto'
Max features	[0.1,1]	Uniform	0.7
Contamination	{'auto',0.01,0.02,0.03,0.04,0.05,0.06,0.07,0.08,0.09,0.1,0.15,0.2}	Choice	0.07
Warm start	{True, False}	Choice	False
Bootstrap	{True, False}	Choice	False
Preprocessor	{None,PowerScale, Standard, Robust, MinMax}	Choice	Robust

For each Machine Learning model applied for the classification tasks, we explored a range of hyperparameters and multiple preprocessors – Supplementary Table 8.

Performance per target definition

We explored how the model performance change with different target definitions, namely by (i) setting a different range for the number of stable weeks to consider that the crisis episode is over, from 1 to 4 weeks, (ii) varying the prediction window (i.e. detecting the next crisis episode within), from 1 to 4 weeks, and (iii) setting a different range for the number of weeks since the prediction was made until the start of the prediction window, from 0 to 2 weeks – Supplementary Table 9.

Supplementary Table 9 XGBoost model results for multiple targets. Values in bold denote the model with the highest performance.

Target	AUROC (std)	AP (std)
Onset after one week without crisis		
Onset within next 7 days	0.796 (0.011)	**0.161 (0.015)**
Onset within next 14 days	0.797 (0.011)	0.161 (0.015)
Onset within next 21 days	0.797 (0.011)	0.160 (0.014)
Onset within next 28 days	**0.797 (0.012)**	0.159 (0.014)
Onset between 7 and 14 days	0.796 (0.012)	0.154 (0.012)
Onset between 7 and 21 days	0.796 (0.012)	0.154 (0.012)
Onset between 7 and 28 days	0.796 (0.013)	0.153 (0.012)
Onset between 7 and 35 days	0.797 (0.013)	0.152 (0.012)
Onset between 14 and 21 days	0.795 (0.014)	0.151 (0.014)
Onset between 14 and 28 days	0.796 (0.015)	0.150 (0.013)
Onset between 14 and 35 days	0.796 (0.015)	0.150 (0.013)
Onset between 14 and 42 days	0.796 (0.015)	0.150 (0.013)
Onset after two weeks without crisis		
Onset within next 7 days	0.787 (0.13)	0.141 (0.015)
Onset within next 14 days	0.788 (0.012)	0.141 (0.014)
Onset within next 21 days	0.788 (0.012)	0.139 (0.014)
Onset within next 28 days	0.788 (0.013)	0.137 (0.013)
Onset between 7 and 14 days	0.787 (0.013)	0.133 (0.012)
Onset between 7 and 21 days	0.787 (0.013)	0.133 (0.012)
Onset between 7 and 28 days	0.787 (0.014)	0.132 (0.012)
Onset between 7 and 35 days	0.788 (0.015)	0.132 (0.013)
Onset between 14 and 21 days	0.786 (0.015)	0.130 (0.014)
Onset between 14 and 28 days	0.787 (0.016)	0.130 (0.013)
Onset between 14 and 35 days	0.787 (0.017)	0.130 (0.013)
Onset between 14 and 42 days	0.788 (0.018)	0.131 (0.013)
Onset after three weeks without crisis		
Onset within next 7 days	0.782 (0.013)	0.131 (0.015)
Onset within next 14 days	0.782 (0.013)	0.131 (0.014)
Onset within next 21 days	0.783 (0.013)	0.130 (0.014)
Onset within next 28 days	0.783 (0.014)	0.127 (0.013)
Onset between 7 and 14 days	0.781 (0.013)	0.123 (0.012)
Onset between 7 and 21 days	0.781 (0.014)	0.121 (0.013)
Onset between 7 and 28 days	0.782 (0.016)	0.120 (0.013)
Onset between 7 and 35 days	0.783 (0.017)	0.120 (0.013)
Onset between 14 and 21 days	0.780 (0.016)	0.119 (0.014)
Onset between 14 and 28 days	0.781 (0.018)	0.119 (0.014)
Onset between 14 and 35 days	0.782 (0.019)	0.120 (0.015)
Onset between 14 and 42 days	0.783 (0.019)	0.120 (0.014)
Onset after four weeks without crisis		
Onset within next 7 days	0.777 (0.014)	0.122 (0.015)
Onset within next 14 days	0.778 (0.013)	0.123 (0.014)
Onset within next 21 days	0.777 (0.014)	0.121 (0.014)
Onset within next 28 days	0.778 (0.015)	0.119 (0.013)
Onset between 7 and 14 days	0.775 (0.014)	0.114 (0.012)
Onset between 7 and 21 days	0.776 (0.015)	0.114 (0.012)
Onset between 7 and 28 days	0.777 (0.017)	0.113 (0.013)
Onset between 7 and 35 days	0.778 (0.018)	0.114 (0.012)
Onset between 14 and 21 days	0.775 (0.017)	0.110 (0.014)
Onset between 14 and 28 days	0.777 (0.019)	0.112 (0.013)
Onset between 14 and 35 days	0.778 (0.020)	0.112 (0.014)
Onset between 14 and 42 days	0.779 (0.021)	0.113 (0.014)

Model interpretability

We analysed dependence plots for the 20 most predictive features for the XGBoost general model according to the mean absolute SHAP values. Six of the dependence plots are included in the main paper, and the remaining fourteen are presented in Supplementary Fig. 1. The dependence plots show the impact that each variable had on the predicted risk score overall the predictions. For instance, the age of the patient carried a positive effect on the risk score if a patient was below 60 and a negative for patients above 60 years. The greatest positive influence of the age on the PRS was observed for the individuals younger than 21 and the greatest negative influence started from the age 73, with a relatively small influence for the patients aged between 21 and 60 years. The total number of crisis episodes experienced by a patient represents the fourth most important feature overall according to the SHAP values. Its influence on the risk scores increases steeply as the number becomes higher, negative for patients who had three crisis episodes and positive for the patients above that threshold. Although with a smaller effect, a similar pattern was observed for the number of years since the first visit, which has a negative influence on the risk score for patients with less than seven years in the system and positive for nine or more years.

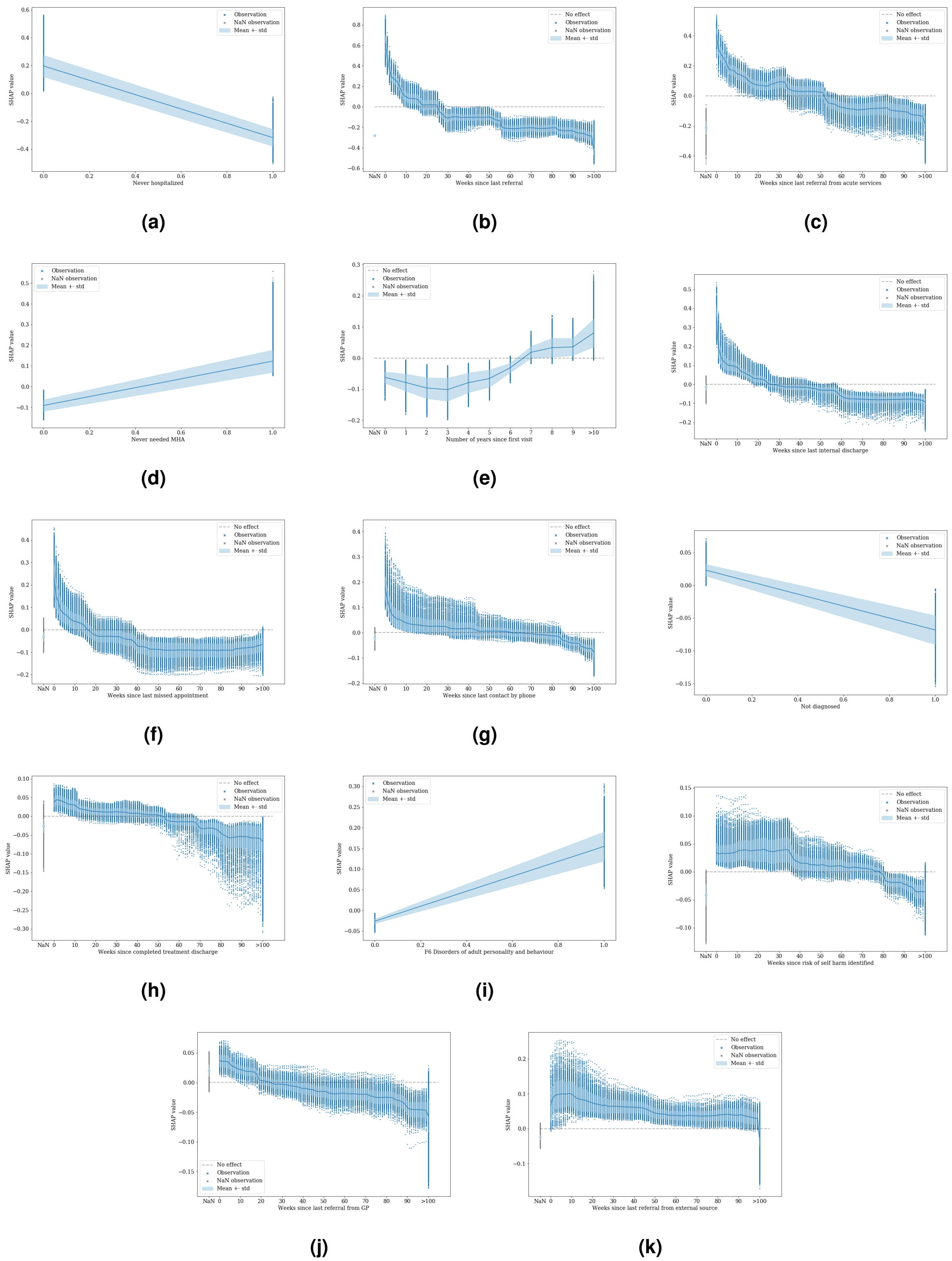

Supplementary Fig. 1. Dependence plots of the most predictive features according to the mean absolute SHAP values. Each plot shows the PRS as the function of different feature values. All samples in the test set are represented by one datapoint, the solid lines and the lighter-coloured envelops represent the mean impact and the standard deviation per feature value, respectively. The variability of each feature value is related to the interaction with the rest of features. Missing values are presented in grey.

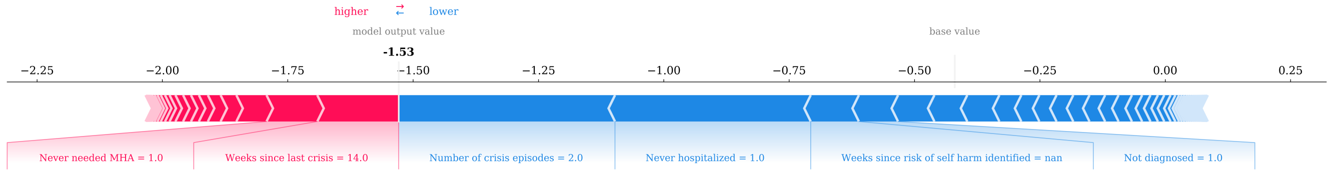

(a)

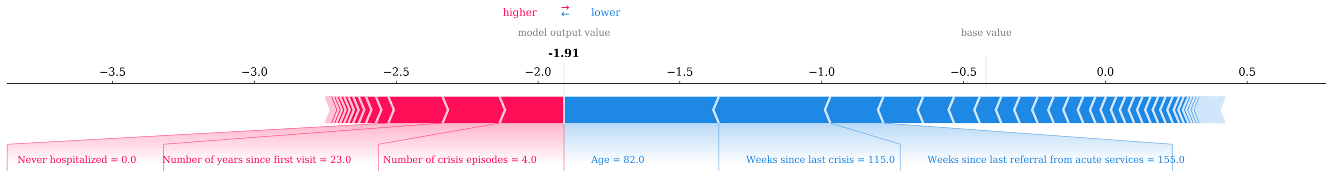

(b)

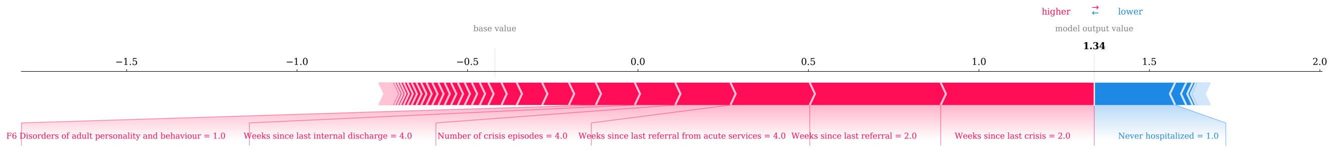

(c)

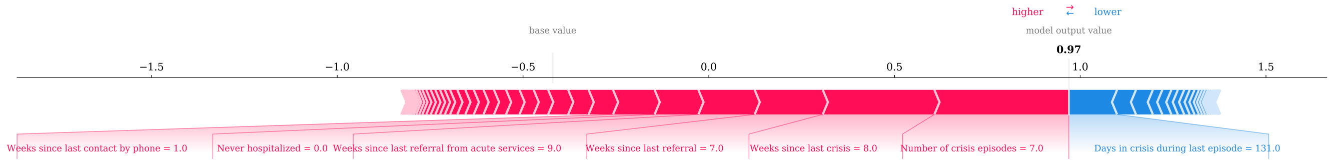

(d)

Supplementary Fig. 2. Examples of features contribution to the predicted risk score. a-d Four representative force plots, depicting how the features contributed to the prediction for four specific data points. **a** Patient not going to have a crisis during the next four weeks (target=0). The model assigned a prediction value of 0.178. **b** Patient not going to have a crisis during the next four weeks (target=0). The model assigned a prediction value of 0.129. **c** Patient going to have a crisis during the next four weeks (target=1). The model assigned a prediction value of 0.792. **d** Patient going to have a crisis during the next four weeks (target=1). The model assigned a prediction value of 0.725.

Supplementary Fig. 2 shows the contribution that each feature had in four different predictions made by the final model. The first two plots correspond to cases in which a patient did not have a crisis (target=0) and the prediction had a value lower than 0.2. The other two correspond to cases in which a patient had a crisis (target=1) and the prediction had a value higher than 0.7. In these four cases, several features contributed either positively or negatively to the final prediction. Both the sign and magnitude of the feature contribution differ in each case. These differences are related to the value of the feature (e.g. weeks since last crisis with a value of 8 has a strong positive effect in Supplementary Fig. 2d and with a value of 115 a strong negative effect in Supplementary Fig. 2b), and the value of the rest of the features (e.g. number of crisis episodes with a value of 4 had a SHAP value of 0.227 in Supplementary Fig. 2b and a SHAP value of 0.162 in Supplementary Fig. 2c). Overall, this analysis exemplifies the complex feature interactions that drive the predicted risk score.

Stability of the most predictive features

To analyse the stability of the most predictive features, we computed the SHAP values from the dataset used to train the model and we ran 100 experiments each with a different randomly generated sample with 40% of the patients.

Supplementary Fig. 3 shows the complete distribution and mean absolute contribution of the 20 most predictive features according to the highest mean absolute SHAP values computed using predictions obtained from the training set, with an AUROC of 0.799 (95% CI 0.796-0.803) and AP of 0.199 (95% CI 0.195-0.203). The 20 most predictive features obtained from the training set corresponded to the same 20 most predictive features obtained from the test set.

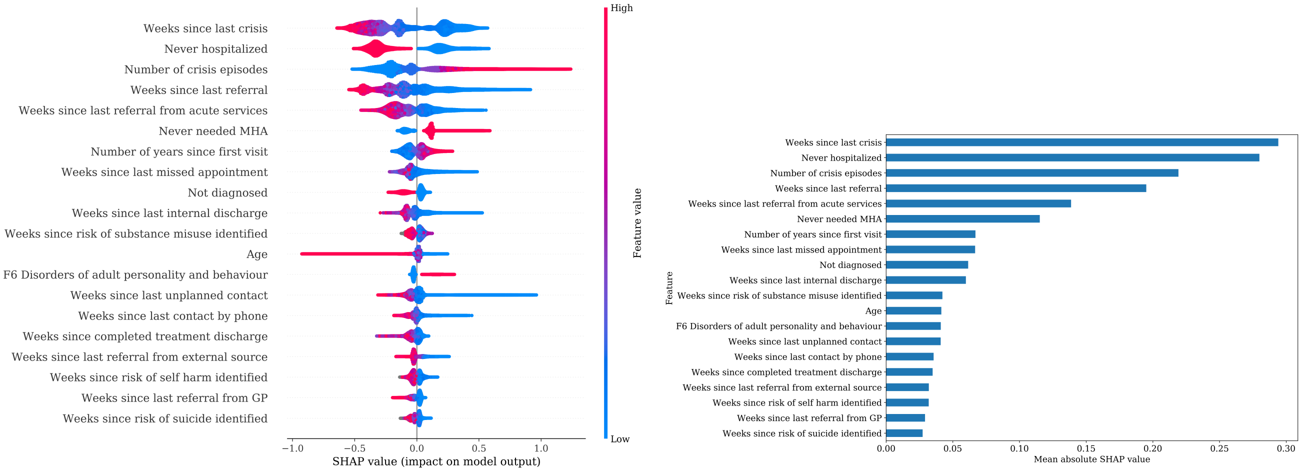

(a) (b)

Supplementary Fig. 3. Most predictive features in the train set a Complete distribution of the SHAP values for the top 20 features based on the highest mean absolute SHAP value in the train set. Each sample of the train set is represented as a datapoint per feature and the x axis shows the positive or negative impact on the model's prediction of the feature. The colour coding depicts the value of the feature and is scaled independently based on its range observed in the data. **b** Absolute feature contribution of the 20 features with the highest mean absolute SHAP value in the train set.

Additionally, we generated 100 different sub-samples by randomly selecting 40% of patients each time. Next, we trained a model for each sub-sample and computed the SHAP values. Finally, we computed the mean absolute SHAP values for each feature per sub-sample and we aggregated the results. Supplementary Fig. 4 shows the 20 most features with the highest mean absolute SHAP value averaged across the 100 samples. This analysis was conducted to further evaluate the stability of the most predictive features across different datasets i.e. selections of patients. We observed that the list of the most predictive features fully match the list of the 20 most predictive features obtained from the test set, although the order among them has changed. Furthermore, 19 among the 20 most predictive features according to the mean absolute SHAP values in the test set matched the list of the 20 most predictive features for more than 70% of the sub-samples. Specifically, "Weeks since the risk of suicide" was found in 47% of the sub-samples. We also quantitatively analysed the consistency of interpretations by computing the cosine similarity of the SHAP values of the top 20 features between the final model and the models trained in each of the samples – resulting in a very high average cosine similarity of 0.987 (std 0.008), with a minimum value of 0.934.

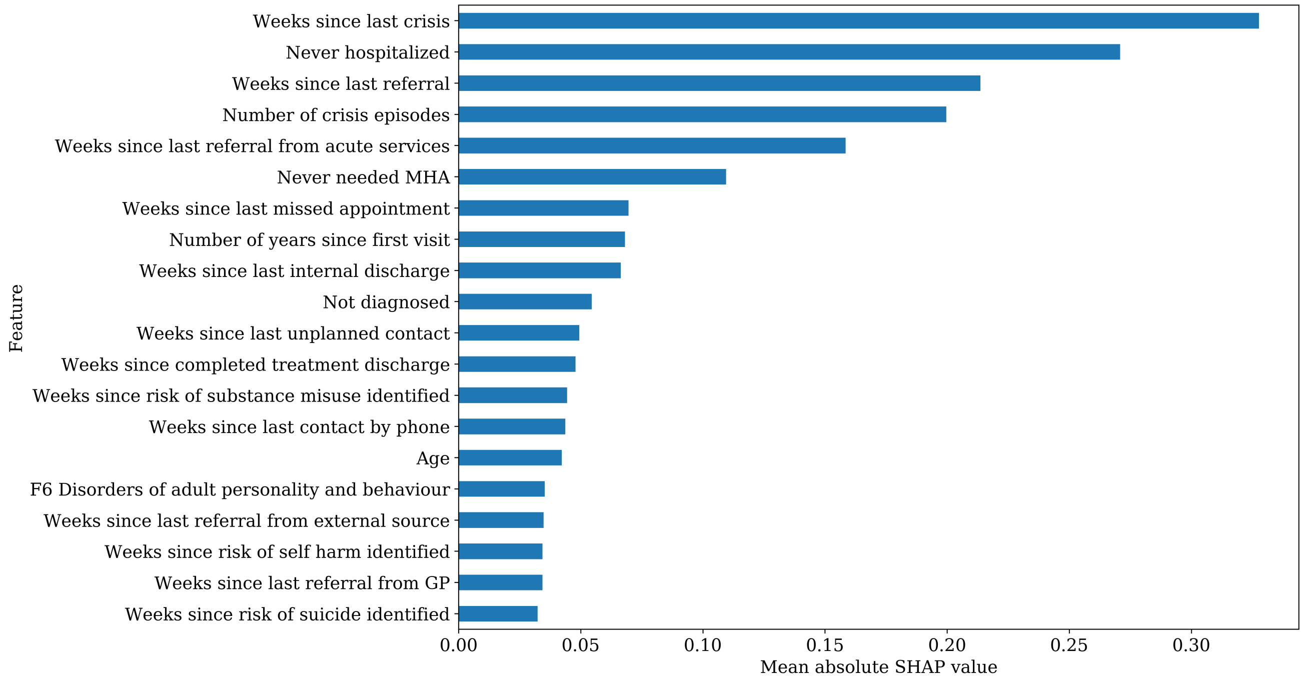

Supplementary Fig. 4. Mean contribution of top 20 features across 100 experiments. Mean absolute feature contribution of the 20 features with the highest mean absolute SHAP value averaged across 100 samples with 40% of the patients each.

Prospective study

Supplementary Table 10 includes the list of clinical indicators that were delivered to the participants (clinicians) in the prospective study alongside the risk score computed for each patient. This information provided more context about each patient flagged by the model – personal characteristics as well as the latest state assessment and interactions with the hospital – thereby the clinicians in decision making and prioritisation.

Supplementary Table 10 List of clinical indicators shown in the decision support tool tested during the prospective study

Clinical indicator	Description
Recent Inpatient: 28 days	Patient had a crisis event during the last 28 days
Recent Inpatient: 2 months	Patient had a crisis event during the last 2 months
Medical Contact: Within 28 days	Patient had a medical contact during the last 28 days
Medical Contact: Within 6 monhts	Patient had a medical contact during the last 6 months
Non-medical Contact: Within 28 days	Patient had a non-medical contact during the last 28 days
PD or Dual Diagnosis	Patient has a co-morbidity of multiple mental disorders
Care Level	Patient is currently assigned to high care level
Care Plan not up to date	Patient has no crisis plan or has not been updated during the past year
Risk Assessment not up to date	Patient has no risk assessment or has not been updated during the past year
DNA without follow up	Patient did not attend last contact and has not been contacted again
FTB or FEP Service User	Patient is in first episode of psychosis or part of an organisation for young people
Aged 65 or above	Patient is 65 years old or older
MHA	Patient is subject to the Mental Health Act (MHA) and its provisions

Qualitative Evaluation

A set of semi-structured interviews was conducted to gain additional insights about the clinical implementation of the crisis prediction model and its effect on decision making in clinical practice. Four themes emerged from the interviews, namely: 1)

Views on implementation and the use of the crisis prediction model; 2) Impact of the predictions on the work of clinicians; 3) Perceived value of the crisis prediction model; 4) Facilitators and barriers for the clinical use.

Views on implementation and the use of the crisis prediction model

All the respondents were in consensus that the training on the use of the model was well executed and useful. The dashboard was perceived straightforward to use, even easier than they had initially expected.

> *It was very useful and I had no problems after I left [the training] in terms of using it as I felt very confident and able to use it*
>
> *The training did not take long at all and it was quick, informative and much easier to do than I assumed it was going to be*

The feedback platform was also easy to use and navigate, despite that some respondents mentioned early technical difficulties (such as information not being saved, inability to open a shared document or misunderstanding on how to correctly open the feedback platform). However, they felt that these issues were addressed promptly.

> *There were a few kind of small technical issues with it...whilst things got up and running but they were quickly resolved and it was easy to use.*
>
> *The tool is not complicated which is good as it would put people off it if there was too much to do.*

The perception of the feedback platform suffered from the expected gaps in the EHRs – some respondents indicated that the information was sometimes out of date and that in such situations patient's notes were the way to acquire more up to date information of patient's status.

> *The scoring criteria used pulls information that can sometimes be out of date.*
>
> *... I know sometimes that is the only thing available to use but sometimes it is a patient you know and you know that there has been other progress notes ... but obviously there is the ability to check patient notes if you wanted to check further.*

This opens two important topics for future research, to mitigate the impact of data gaps in the EHRs. Firstly, the information from unstructured data (i.e., patient notes) can be incorporated in the prediction model, secondly, the patient notes can be automatically processed to extract a summary for the dashboard presented to clinicians.

Impact of the predictions on the work of clinicians

Respondents indicated that the tool was used as an additional resource in their workflow and that it did not require extensive additional work. Interestingly, they suggested that the number new cases to review introduced by the algorithm was well distributed per clinician but that a potentially increased number of flagged cases would have exceeded their capacity.

> *It is manageable as it is but if the cases reviewed increased, people would be annoyed and would likely have less time to spend on cases.*

Others welcomed the increase in the cases flagged every two weeks but believed more clinicians from other disciplines needed to participate in the project.

> *If that [number of flagged cases] would be increased, I think that is fine but other disciplines need to be involved in this, it does not need to be just nurses but doctors, psychologist, occupational therapists, Students and support workers. I think a lot more people can be a part for this and they are not.*

Respondents described types of actions they took in response to a patient being flagged by the prediction algorithm. They contacted many patients by telephone, they made home visits, rearranged visits, brought forward outpatient appointments and/or reviewed patients in multidisciplinary meetings. Some of the examples included:

There has been a few times when the patients that come up have been a bit of a surprise and then I thought I should be more alert to them and making sure I see them a bit more regularly or see them as soon as I can by moving appointments.

Sometimes I have asked GPs to give them a telephone call or taken cases to MDTs for discussion... which is always a good place to put them in as medics will bring the outpatients appointments forward or put them on the care coordinators waiting list which is positive.

One respondent mentioned that there were instances when they did not understand after reviewing cases why some of the patients were on the list therefore they did not follow up and the patient subsequently went into crisis which made them realise that they needed to be more thorough in their reviewing process.

There was a patient that was allocated to me that is in hospital now but when I reviewed her, I did not understand why she was on there [in the dashboard flagged by the algorithm] so did not act on this but on my next follow up with her, I had to call an ambulance.

There has been many times when patients have been missed in the service, maybe they did not turn up for their doctors appointment and another appointment was not sent out or when there has been contact with our duty team where there were signs of relapse but this has not been picked up on or escalated, the tool is very helpful to identify these cases which would have otherwise been followed up on.

Perceived value of the crisis prediction model

As per design, the dashboard was presenting patients with the highest risk of experiencing a crisis. This implied the inclusion of patients whose risk had been already managed, which was unexpectedly reflected in the clinicians' ratings, and it also came up in the interviews.

We have found a lot of value in some of the cases...but a lot of the cases we are coming across, we know about… especially care coordinator would be seeing them regularly and will have very up to date information on what is going on ...

The tool is more useful for patients not under care coordination as patients on care support tend to see a doctor every 3 to 4 months and in between they do not see anyone and we are relying on them making contact if they are unwell"

Nevertheless, respondents highlighted that even in such cases the tool could help in managing caseload priorities – namely, by providing additional information to revise cases more thoroughly, by serving as a reminder, and even by identifying cases that have been lost due to miscommunication or gaps in the system.

I think it is good in terms of a manager's perspective, it helps care coordinators look more thoroughly at their cases and as you know with busy jobs, it is easy to forget things at times and it acts as a reminder.

Sometimes even if the information of a patient should be related to us there sometimes are communication issues because people are busy... so we may lose that one person in crisis and nobody remembered to tell another person about it...this is sometimes picked up by the tool.

Importantly, even though most of the flagged cases were already under supervision, many (correctly flagged) cases were unknown to the clinicians. This was recognised as an important value in clinical practice as it brings an opportunity to prevent or mitigate crises – otherwise these patients would have been "lost in the system".

It highlights people that would otherwise, in my opinion, get lost in the system.

The tool is useful in flagging up people who need help that have gone missed or unnoticed before going into crisis.

In a similar line, some respondents mentioned that the tool had not influenced how they perceived risk but that it shed the light onto some patients that they would not have known about or patients in need that would have gone unnoticed.

It has not changed how I view risk and I do not think more of about risk, as I think of risk all the time… as a clinical lead, I am continuously thinking about how to reduce the risk to someone. I think we would lose, if we did not have this in place, a lot of people who would slip through the net .

Overall, it was encouraging that all respondents believed that the tool should be embedded in their everyday working practice. They expressed their interest to carry on using the tool as business as usual.

I definitely think it should be incorporated as part of our normal working practice because I think it has shown, and everyone I have looked at I found informative, and of course there are some that I think I know this person and they won't go into crisis and they will be fine but others where I have reviewed and I thought oh my goodness, I can't believe this person has never had any contact and we would never have known and so I believe that it should be used in everyday practice.

Facilitators and barriers for the clinical use

The adoption of any new tool, technology or a process can be challenging. Most respondents mentioned that the adoption of the crisis prediction tool was greatly facilitated by digital champions and clinical leads for the project who provided support and directly addressed a any problem or a concern.

It was helpful to have a lead to better explain things that we didn't understand and also to relay issues to when there were issues with the system like logging in and saving responses.

The training was very helpful and then having the lead was helpful as he is on site at least once or twice a week to help people if they need it. So if there has been any questions I can ask him then or email him and he will get back to me quickly so that kind of resolved any issues quickly.

Some respondents voiced concerns, both in the initial phase and during the prospective study, about the responsibility of using this tool. Although beyond the scope of this manuscript, this represents a crucial challenge to address before planning a wider implementation.

I think initially it was just what if we don't call the patient that we have been allocated to... if we don't see or speak to them and if anything happened to them, whether that would be seen as the fault of the clinician because that was one of the patients we were looking at that week and if this is rolled out, and not just a trial, that was going be like an everyday practice worry.

Time constraints to revise the new cases is another important aspect to consider for the practical implementation. Most of the respondents indicated that an increase in workload to review additional cases was properly designed, but a few participants reported difficulties.

… if the patient is somebody you are already care coordinating, they are on your case load anyway which is fine but if it is not, then you end up having to make calls to people or seeing them face to face and following up which takes a lot of time.

I do find value in it but I will admit that sometimes when I am really busy because of the time frame to get things done I feel like I am rushing it more than other times so probably not getting as much value of it when I'm rushing it. But when I can take the time, not that it's very time consuming, it's just the nature of the job that it's so busy sometimes you can't even use the toilet, which is typical. So it is not that I feel this is time consuming in any way and it is very easy to use. But when I use it properly I find it very beneficial.

References

1. Barocas, S., Hardt, M. & Narayanan, A. *Fairness and Machine Learning* (fairmlbook.org, 2019). http://www.fairmlbook.org.

2. Lloyd, T. *et al.* Incidence of bipolar affective disorder in three uk cities: Results from the Æsop study. *Br. J. Psychiatry* **186**, 126–131, DOI: 10.1192/bjp.186.2.126 (2005).

3. Qassem, B. P. S. N. e. a., T. Prevalence of psychosis in black ethnic minorities in britain: analysis based on three national surveys. *Soc. Psychiatry Psychiatr. Epidemiol.* (2015).

4. Schwartz, R. C. & Blankenship, D. M. Racial disparities in psychotic disorder diagnosis: A review of empirical literature. *World journal psychiatry* **4 4**, 133–40 (2014).

5. Zafar, M. B., Valera, I., Rogriguez, M. G. & Gummadi, K. P. Fairness constraints: Mechanisms for fair classification. In *Artificial Intelligence and Statistics*, 962–970 (PMLR, 2017).

6. Zehlike, M., Hacker, P. & Wiedemann, E. Matching code and law: achieving algorithmic fairness with optimal transport. *Data Min. Knowl. Discov.* **34**, 163–200 (2020).

7. Skeem, J. L. & Lowenkamp, C. T. Risk, race, and recidivism: Predictive bias and disparate impact. *Criminology* **54**, 680–712 (2016).
